# Synthesis of Bis-tetraphenylethene as a Novel Turn-On Selective Zinc Sensor

**DOI:** 10.1021/acsomega.3c02955

**Published:** 2023-07-03

**Authors:** Abdullah Saleh Hussein, Ferruh Lafzi, Haydar Kilic, Sinan Bayindir

**Affiliations:** † Department of Chemistry, Faculty of Sciences and Arts, Bingöl University, Bingöl 12000, Türkiye; ‡ College of Education Chemistry Department, Salahaddin University—Erbil, Erbil 44002, Iraq; § Department of Chemistry, Faculty of Sciences, Atatürk University, Erzurum 25240, Türkiye

## Abstract

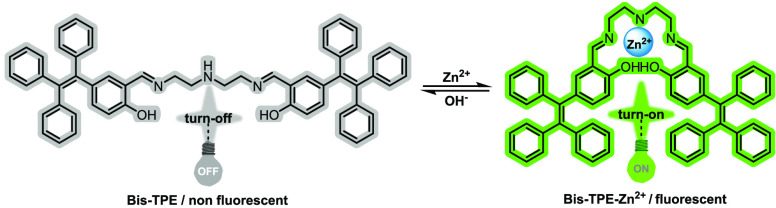

The main purpose of this study is the synthesis of novel fluorescent **Bis-TPE** and the investigation of its wide range of photochemical behaviors. For this purpose, initially, **Bis-TPE** was synthesized. Following this, the interactions of **Bis-TPE** with a wide range of ions were studied in EtOH using ultraviolet–visible (UV–vis) and fluorescence spectroscopy. As a result of all UV–vis and fluorescence studies, it was determined that **Bis-TPE** showed turn-on sensor features against Zn^2+^ ions. Moreover, the limit of detection (LOD) and *K*_a_ values of Bis-TPE/Zn^2+^ were calculated as 0.97 μM (970 nM) and 3.76 × 10^5^ M^–1^, respectively. Moreover, all reversal studies resulted in switchable on/off variation of the alternative addition of ZnCl_2_ and [Bu_4_N]OH to **Bis-TPE**. This result also implies that the probe **Bis-TPE** also exhibits specific OH^–^ sensor properties in the presence of zinc.

## Introduction

**Figure 1:**
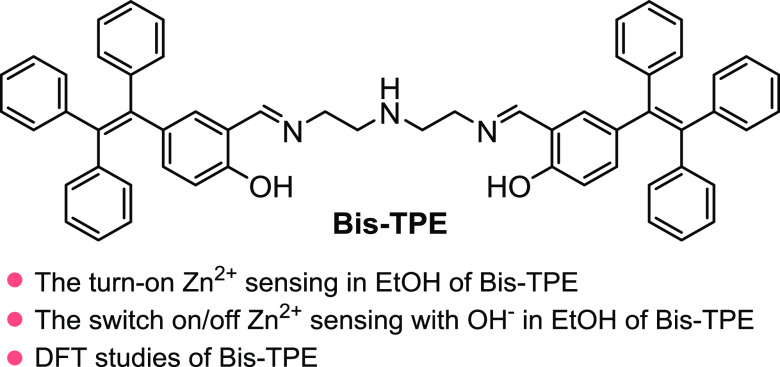
Strategies for examination of recognition properties of **Bis-TPE**.

Nowadays, instead of methods that require expensive instrumentation and large volumes of samples, such as voltammetry, plasma mass, and atomic emission spectrometry, colorimetric and fluorometric detection of ions is of great interest due to its simplicity and sensitivity.^1−10^ Various metal particles take up an essential part of our daily physiological life. Zinc (Zn) ions, one of these metals, are the second most abundant element in the human body after iron ions.^11−13^ Various biological catalysts found in living systems contain zinc.^14^ For example, zinc is an abundant trace element in red blood cells as a key component of the enzyme carbonic anhydrase (hCAs), which aids in carbon dioxide metabolism pathways.^15,16^ In addition, the brain, muscles, bones, prostate, retina, kidneys, and liver are also organs that contain the most zinc.^17,18^ However, both the deficiency and excess of zinc ingredients in the living body need to be controlled carefully. Because abnormal excess levels of zinc, such as deficiency of zinc, can cause various diseases including Alzheimer’s disease, brain diseases, Parkinson’s disease, hair loss, etc.^19−21^ Hence, monitoring zinc specifically at all levels (especially μM) is of great importance in terms of living healthily, and the generation of efficient chemosensors specific for zinc detection is an important scientific necessity. Speaking of the importance of the specific detection of ions, nowadays, another technologically important and quite remarkable issue is aggregation-induced emission (AIE).^22^ In this context, tetraphenylethene (**TPE**) as an effective fluorophore has received much attention since the beginning of this century. The TPE unit can also function as an energy donor part in combination with some other fluorophores reported in many pieces of literature. In addition, organics containing TPE units have been used as effective organic sensors in recent years.^23−34^ In this context, we primarily synthesized novel bis-tetraphenylethene (**Bis-TPE**). Following synthesis, the AIE studies and detection properties against a wide range of metals and anions of **Bis-TPE** were investigated by colorimetric and spectroscopic techniques (Figure 1).

## Results and Discussion

### Chemistry

**Figure 2:**
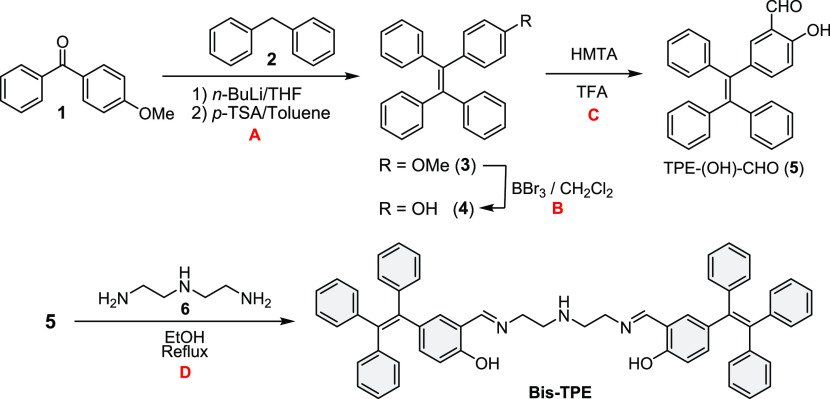
Synthesis strategy of **Bis-TPE**.

The synthesis of TPE-based organic probes and investigation of ion sensor properties is a popular phenomenon from the past to the present. For this purpose, we synthesized novel **Bis-TPE** over multistep reaction routes (Figure 2). In this context, initially, output compounds TPE-(OMe) (**3**), TPE-(OH) (**4**), and TPE-(OH)-CHO (**5**) were obtained, as stated in the related literature (Figure 2A–C).^35,36^ Following the synthesis of output molecules **3**, **4**, and **5**, the target organic probe novel **Bis-TPE** was synthesized from the reaction of TPE-(OH)-CHO (**5**) with *N*1-(2-aminoethyl)ethane-1,2-diamine (**6**) in ethanol at the reflux temperature of ethanol (Figure 2D).

### Ultraviolet–Visible (UV–Vis) and Fluorescence Response of Bis-TPE to Various Ions

**Figure 3:**
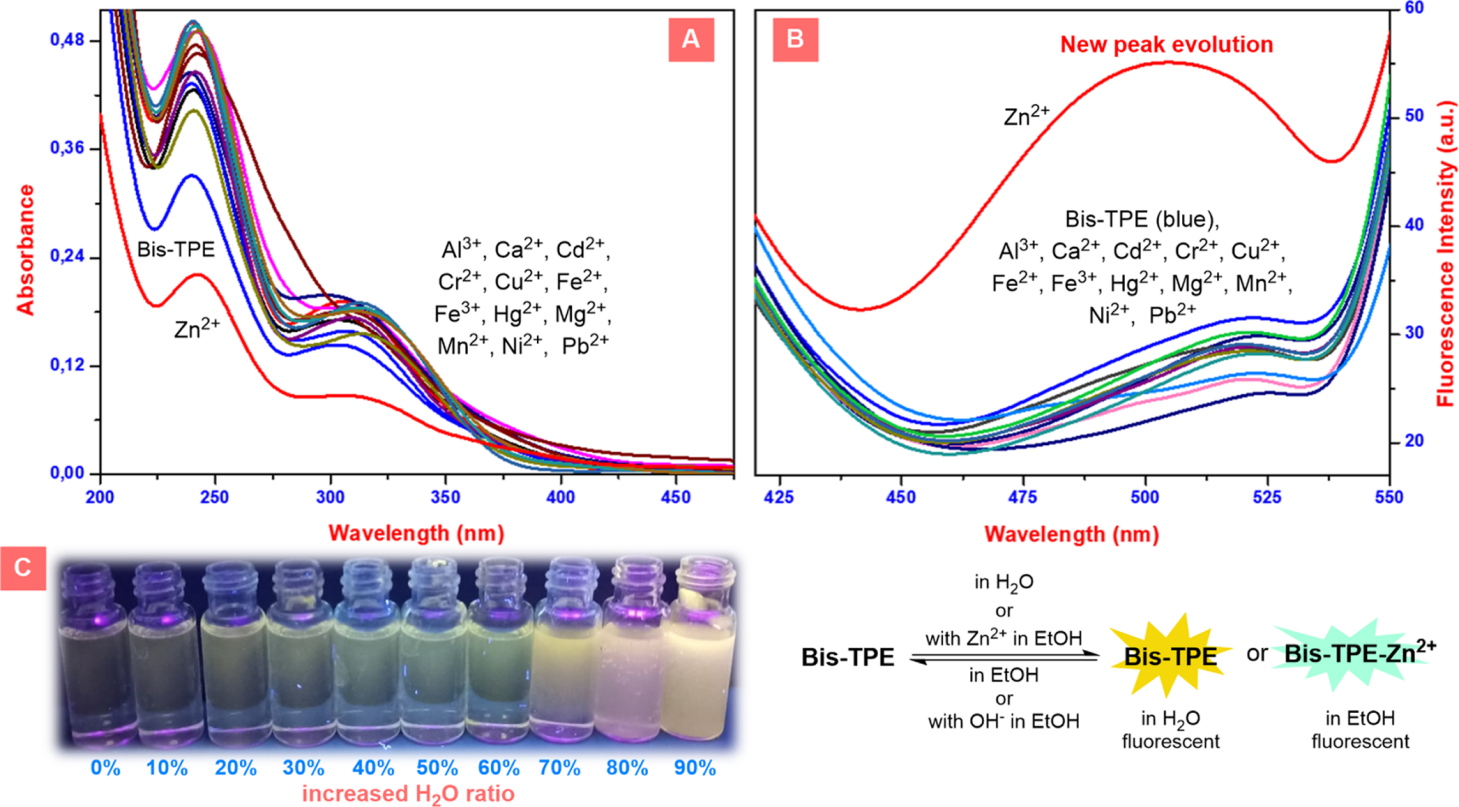
UV–Vis (A) and fluorescence (B) spectra of **Bis-TPE** in the absence and presence of metal ions in EtOH and (C) the photographic images of **Bis-TPE** in the various H_2_O ratios.

Following the synthesis of **Bis-TPE**, to clearly investigate the interaction of the probe with a variety of ions (cations and/or anions), the ultraviolet–visible (UV–vis) and fluorescence studies of **Bis-TPE** were first performed in a variety of solvent systems such as MeOH, EtOH, CH_3_CN, dimethyl sulfoxide (DMSO), and tetrahydrofuran (THF) and their aqua solvent systems (Figure S5). While no interaction was observed in aqueous solutions, similar results were obtained from studies performed in other organic solvents such as CH_3_CN, DMSO, and THF. On the other hand, although similar results were obtained with EtOH in the studies carried out in MeOH, ethanol was preferred as a solvent, considering both the lower fluorescence intensity and the harmful effect of methanol on health (Figure S5). In this context, studies were carried out in EtOH, which is relatively less harmful in related solvents. The UV–vis spectra for all ions were recorded after about 10 min of the addition of 2 equiv of each mentioned ion, and the UV–vis spectra were obtained as given in Figure 3A. As illustrated in Figure 3A, the UV–vis spectrum of **Bis-TPE** exhibited a broad band at 305 nm and also a strong band at about 231 nm. The interaction of **Bis-TPE** with varieties ions of metals (Al^3+^, Ca^2+^, Cd^2+^, Co^2+^, Cu^2+^, Fe^2+^, Fe^3+^, Hg^2+^, Mg^2+^, Mn^2+^, Ni^2+^, Pb^2+^, and Zn^2+^ as their chloride salts) and anions ([Bu_4_N]F, [Bu_4_N]Cl, [Bu_4_N]Br, [Bu_4_N]I, [Bu_4_N]AcO, [Bu_4_N]BnO, [Bu_4_N]HSO_4_, [Bu_4_N]ClO_4_, [Bu_4_N]CN, [Bu_4_N]SCN, [Bu_4_N]H_2_PO_4_, [Bu_4_N]OH) was investigated in EtOH. Except for minor changes, no significant changes were observed from the absorption studies of **Bis-TPE** with anions (Figure S6). According to UV–vis studies in EtOH, in the presence of Zn^2+^, the absorption band of **Bis-TPE** at 231/305 nm decreased to 240/310 nm with red shifts. On the other hand, in UV–vis studies of **Bis-TPE** with other metal ions in EtOH, it was observed that the absorption band of **Bis-TPE** at 231/305 nm increased to 225–230/301–310 nm with blue–red shifts (Figure 3A). In addition to UV–vis studies, fluorescence spectroscopy studies were also carried out in order to measure the ability of **Bis-TPE** (10 μM) as a fluorescent ion (cation and/or anion) sensor. In parallel with the UV–vis studies, no significant and specific changes were observed from the interaction of **Bis-TPE** with anions (Figure 3B). From fluorescence studies with cations, a specific spectral change was observed against Zn^2+^ ions, again in agreement with UV–vis studies. Thus, to gain further insight into the selective and sensitive Zn^2+^ binding ability of **Bis-TPE** (10 μM) toward a series of cations were evaluated by observing changes in their fluorescence emission spectra in ethanol (Figure 3B). As illustrated in Figure 3B, **Bis-TPE** alone exhibited two fluorescence peaks at 382 and 570 nm in EtOH with an excitation of 280 nm (Figures 3B and S7A). Under the same conditions, the fluorescence spectra of the interactions of **Bis-TPE** with metal ions were also measured within about 10 min after the addition of ions. As a result of **Bis-TPE** interactions with metal ions, very small changes (approximately 1 nm) were observed in other metal ions except for Zn^2+^ ions, while important changes were observed due to the interaction of **Bis-TPE** with Zn^2+^ ions. That is, it was determined that the fluorescence peaks of **Bis-TPE** at 503 nm increased in the presence of Zn^2+^ (Figure 3B, red). Consistent with UV–vis studies, it is seen that the fluorescence peak of **Bis-TPE** at 503 nm increased with Zn^2+^ and decreased due to interactions with others. As a result of the studies, it can be said that **Bis-TPE** can be a specific turn-on fluorescent sensor at 503 nm for Zn^2+^ ions. Another point worth noting here is the nature of the fluorescence peak around 570 nm. Namely, whether this peak is a fluorescence signal or a 2nd-order diffraction of the excitation light. So here, the aggregated particles enhance the light scattering reasonably, and this may be checked by excitation wavelength dependency. The wavelengths of the monochromatic light from the monochromator for excitation were set at 280, 300, 320, 340, 360, 380, 420, 440, and 460 nm (Figure S7B). Fluorescence is most efficiently excited at 280 nm, and the fluorescence intensity decreases continuously to λ_ex_ of 460 nm. The blue fluorescence intensity clearly decreases significantly as the excitation wavelength increases, and this is consistent with numerous previously reported fluorescence intensity results.^37−39^ Following extensive anion studies, in this stage, AIE studies of **Bis-TPE** were also carried out with increasing water content in ethanol. The colorimetric studies show that the color change shifted from colorless to bright orange with increasing water content. Additionally, the fluorescence intensity of the TPE-based organic probe **Bis-TPE** started to increase in small intervals with the addition of 10% of H_2_O and reached its maximum value at 70–90% of H_2_O. That is, the fluorescence intensity at 575 nm of **Bis-TPE** in EtOH/H_2_O (v/v) has increased with increasing water percentage. This may be essentially a clear indication that **Bis-TPE** exhibits an AIE feature. However, the fact that **Bis-TPE** excited at 280 nm gave fluorescence intensity at 575 nm suggests that this is not a fluorescence signal, as in anion studies, but may instead be a 2nd-order diffraction. Indeed, it is thought-provoking what could be the reason why it does not give significant results in terms of fluorescence but gives a visible color change with the increasing water ratio. Here, it is thought that the AIE behavior of **Bis-TPE**, which contains nucleophilic groups such as nitrogen (N) and oxygen (O), is the suppression of the photoinduced electron transfer due to the increased aggregation of **Bis-TPE** (Figures 3C and S8). Additionally, the excited state intramolecular proton transfer (ESIPT) process is influenced by the acidity of the proton donor and the alkalinity of the proton receptor, which depends on the charge distribution of the donor and receptor atoms. In this context, the UV–vis and fluorescence properties of **Bis-TPE** suggest that it undergoes the ESIPT process more readily due to a significant change in electron density in its excited state. The fluorescence emission phenomenon may be caused by the tautomerization of **Bis-TPE** through the ESIPT process, where a proton from the OH group shifts to the CH=N group via an intramolecular hydrogen bond in the excited state (Scheme S1).^40^

**Figure 4:**
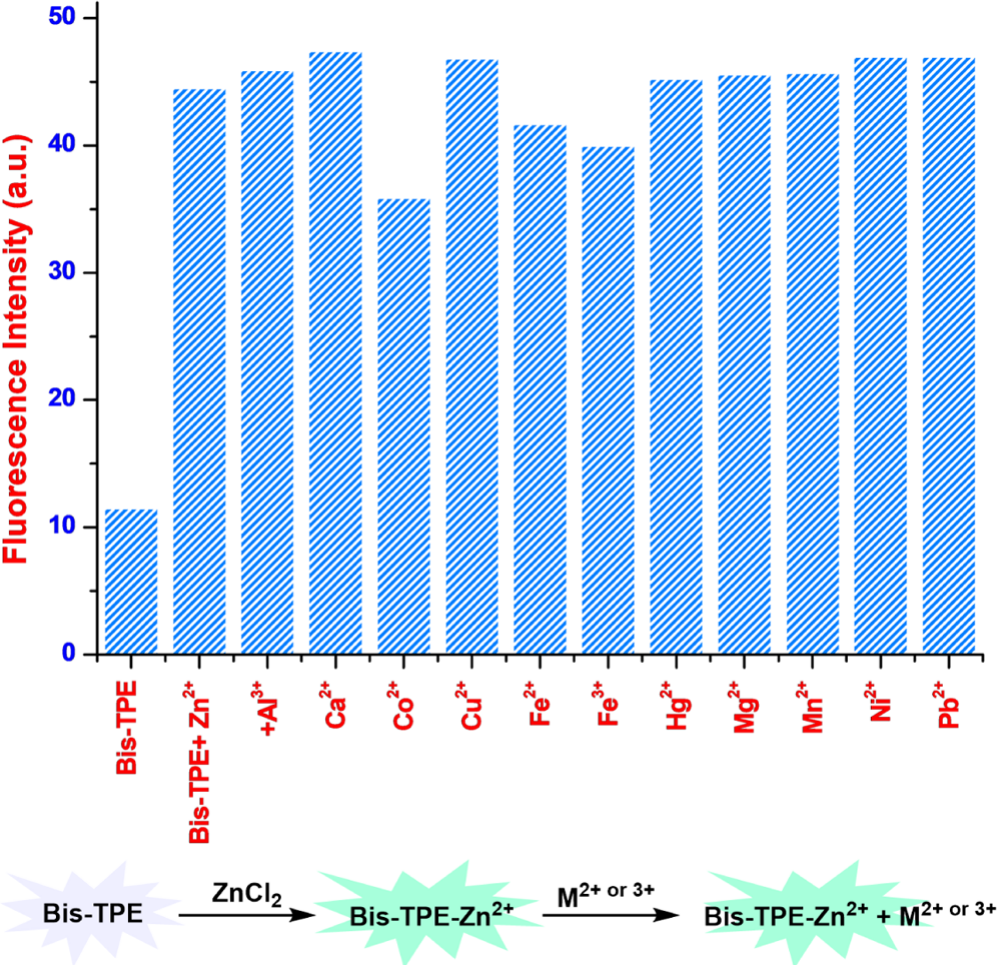
Selectivity of **Bis-TPE** for zinc in the presence of other metal ions.

In addition to UV–vis and fluorescence experiments, the individual interactions of probes with ions and the specific spectral changes as a result of these interactions are also important due to the fact that it is unlikely that a single metal will always be present in samples. In this context, at this stage, following UV–vis and fluorescence experiments (Figure 3), to study the influence of other metal ions on Zn^2+^ ions binding with **Bis-TPE**, competitive experiments in the presence of ZnCl_2_ with other metals were performed in ethanol (Figures 4 and S9). The results of experiments showed that fluorescence reduction induced by the mixture of Zn^2+^ with all other metal ions was like that induced by zinc alone in EtOH (Figure 4). Thus, as a result of all of these studies, none of the other tested metal ions were found to interfere with the interaction of **Bis-TPE** with Zn^2+^ in ethanol.

**Figure 5:**
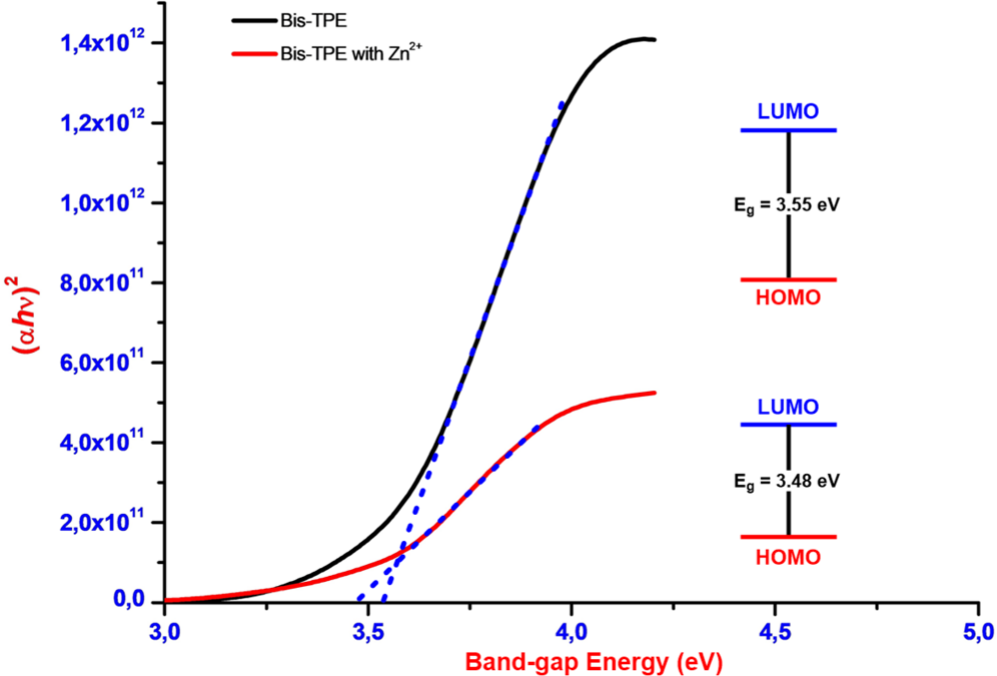
Band-gap energies of **Bis-TPE** and Bis-TPE-Zn^2+^.

Following the experimental UV–vis studies, the band-gap energy (*E*_g_) values of **Bis-TPE** and **Bis-TPE-Zn**^**2+**^ were also determined experimentally (Figure 5). For this, first, the absorption coefficient (α) was calculated using the formula 1. Here, *d* indicates the film thickness and *T* indicates the percent optical transmittance value.
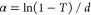
1Then, the *E*_g_ range of **Bis-TPE** and **Bis-TPE-Zn**^**2+**^ were calculated using the formula 2. Here, (*h*ν) is the photon energy and *K* is the material constant.^41^
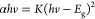
2Moreover, the *E*_g_ can be correlated with electrical conductivity^42^ and kinetic stability. An organic molecule with a wide highest occupied molecular orbital (HOMO)–lowest unoccupied molecular orbital (LUMO) energy gap is considered to have high chemical hardness and good stability, while a molecule with a narrow HOMO–LUMO energy gap is considered to have good chemical reactivity. For this purpose, at this stage, the experimental calculation of *E*_g_ values was calculated. The *E*_g_ values of **Bis-TPE** and **Bis-TPE-Zn**^**2+**^ were calculated as 3.55 and 3.48 eV, respectively. Although the numerical values are different, the experimental and theoretical results are proportionally compatible with each other. Additionally, according to the experimental *E*_g_ values and the obtained absorbance (240/310 nm) measurements, **Bis-TPE** can also be used in photovoltaic applications were predicted.

**Figure 6:**
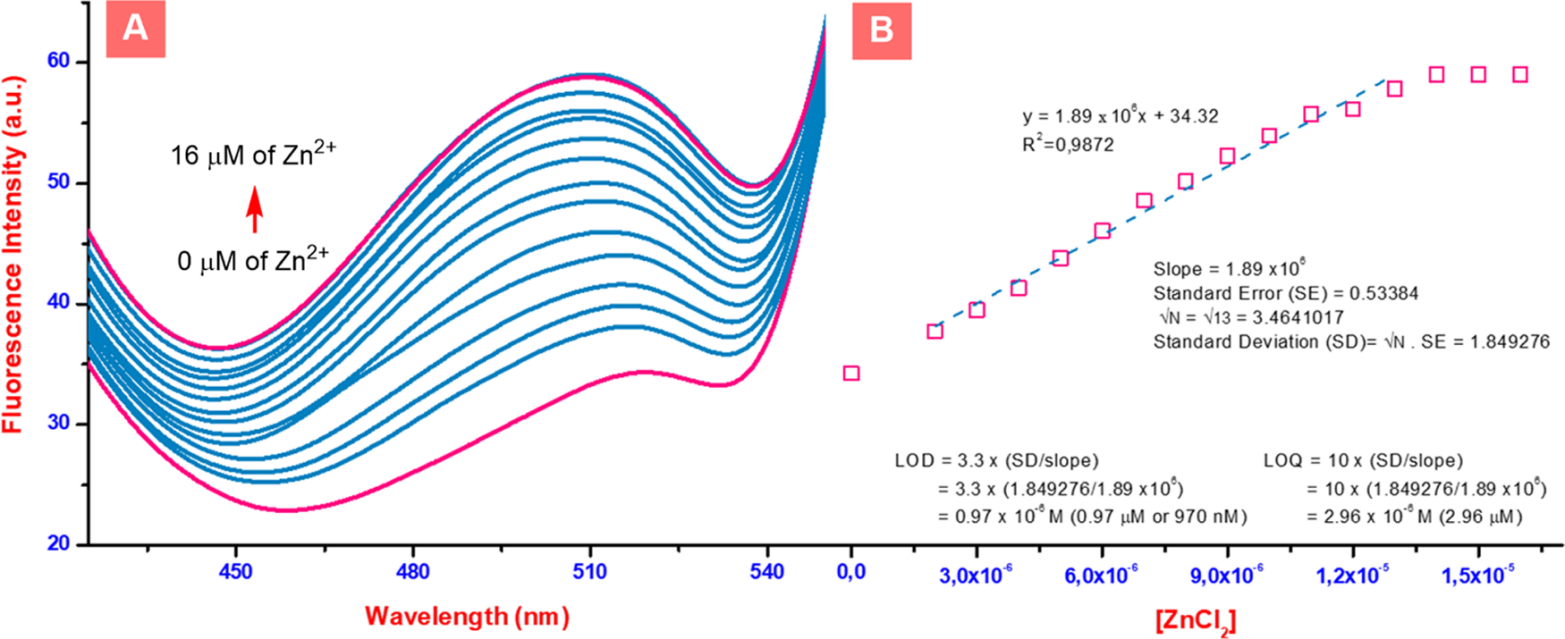
(A) Fluorescence spectra of **Bis-TPE** in the presence of Zn^2+^ and (B) the change in the fluorescence intensity of **Bis-TPE** with the increasing concentration of Zn^2+^.

Following general UV–vis and fluorescence experiments of **Bis-TPE** with selected ions to study the sensitivity of **Bis-TPE** toward Zn^2+^ ion sensing, the fluorescence reply of the interaction of **Bis-TPE** with increasing ZnCl_2_ with excitation at 280 nm in EtOH was examined (Figure 6A). Upon the progressive addition of ZnCl_2_, the fluorescence intensity gradually increased. In the presence of about 16 μM of ZnCl_2_, the fluorescence difference between **Bis-TPE** and the Bis-TPE-Zn^2+^ ion was more than three times of **Bis-TPE**. As more ZnCl_2_ was added, the fluorescence intensity of **Bis-TPE** increased with the concentration of Zn^2+^ ions. Following this, the limit of detection (LOD) and the limit of quantitation (LOQ) values of **Bis-TPE** for Zn^2+^ ions were calculated by the fluorescence titration results. The LOD and LOQ values were calculated from the relevant formulas (Figure 6B), and the LOD and LOQ values of **Bis-TPE** for Zn^2+^ ion were calculated as 0.97 μM (970 nM) and 2.96 μM in EtOH, respectively (Figure 6B).

**Figure 7:**
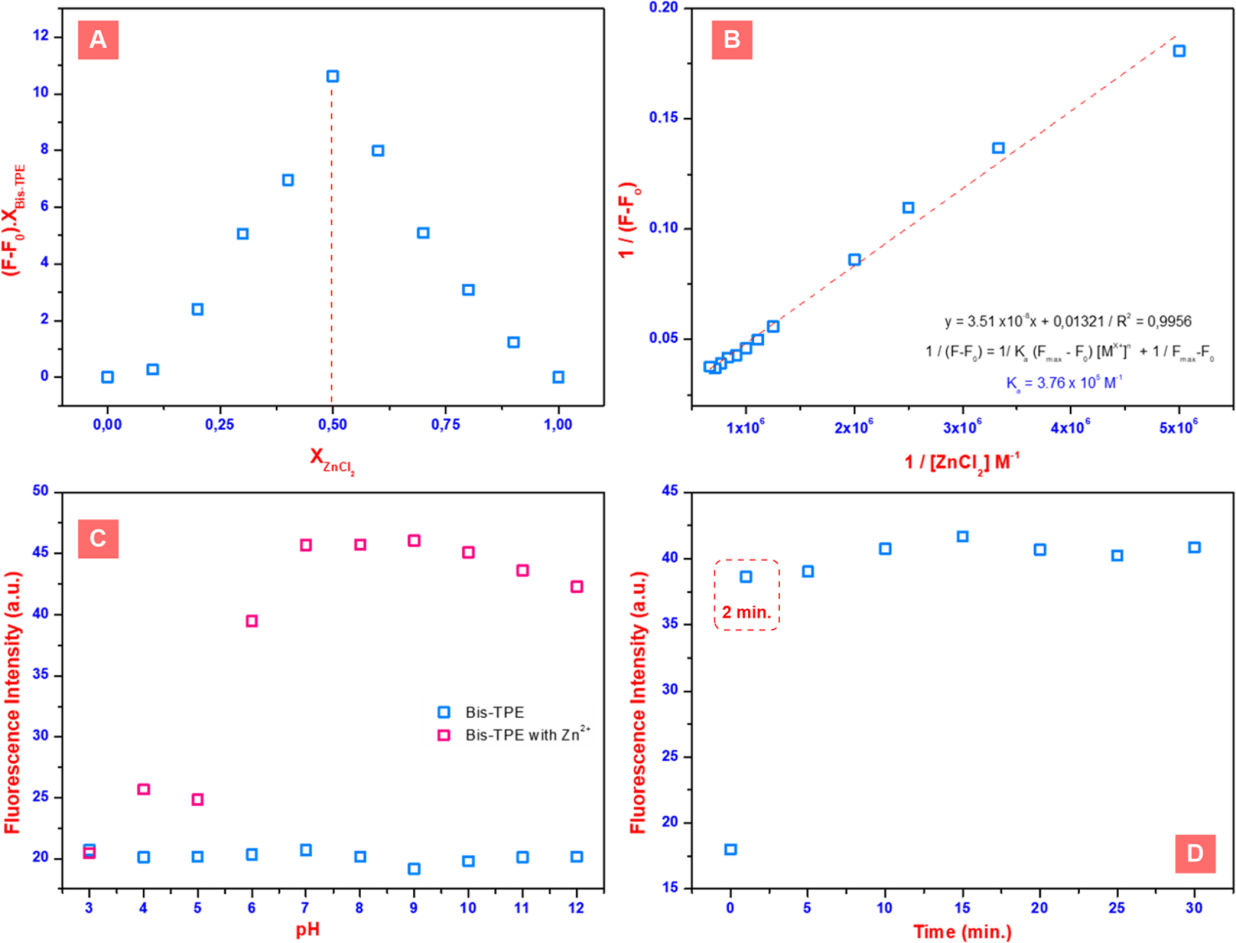
(A) Job’s plot of **Bis-TPE** with ZnCl_2_, (B) the Benesi–Hildebrand plot based on a 1:1 association stoichiometry between **Bis-TPE** and Zn^2+^, (C) the fluorescence values of **Bis-TPE** with [ZnCl_2_] at different pH (3–12) values, and (D) the exposure times of **Bis-TPE** with ZnCl_2_.

The calculation of the binding constant (*K*_a_) value is also a very important parameter for characterizing sensor candidates. To calculate *K*_a_ values, first, the stoichiometry of the binding between the zinc and the organic probe **Bis-TPE** needs to be determined. To determine the binding modes and stoichiometry of **Bis-TPE** with ZnCl_2_, the Job’s plot analysis was carried out (Figure S10) as in the Supporting Data file, and the interaction ratio between zinc and **Bis-TPE** was calculated to be 1:1 (Figure 7A). Following this, the *K*_a_ value of **Bis-TPE** for Zn^2+^ was calculated by the fluorescence titration results and relevant formula, as mentioned in Figure 6B. According to the calculation, the *K*_a_ value of **Bis-TPE** with Zn^2+^ was calculated to be 3.76 × 10^5^ M^–1^ (Figure 7B), and this result implies that there is a relatively strong binding between the probe **Bis-TPE** and zinc ions. Similarly, the pH and time studies are also important for the characterization of sensor candidate organic probes. First, pH studies were performed in ethanol over a wide range from 3 to 12 (Figures 7C and S11). As a result of pH studies, it was observed that **Bis-TPE** had maximum fluorescence values at pH 6–12, while it had relatively lower fluorescence values at pH 3–5 (Figure 7C, pink). Additionally, no significant difference is observed except for minor changes at different pH values of **Bis-TPE** (Figure 7C, blue). On the other hand, the fluorescence intensity of **Bis-TPE** exposed to Zn^2+^ was monitored as a function of exposure time (Figures 7D and S12). As can be seen in Figure 7D, the initial fluorescence intensity of **Bis-TPE** at 503 nm decreased dramatically upon exposing (about 2 min) **Bis-TPE** to the Zn^2+^ ions at room temperature.

**Figure 8:**
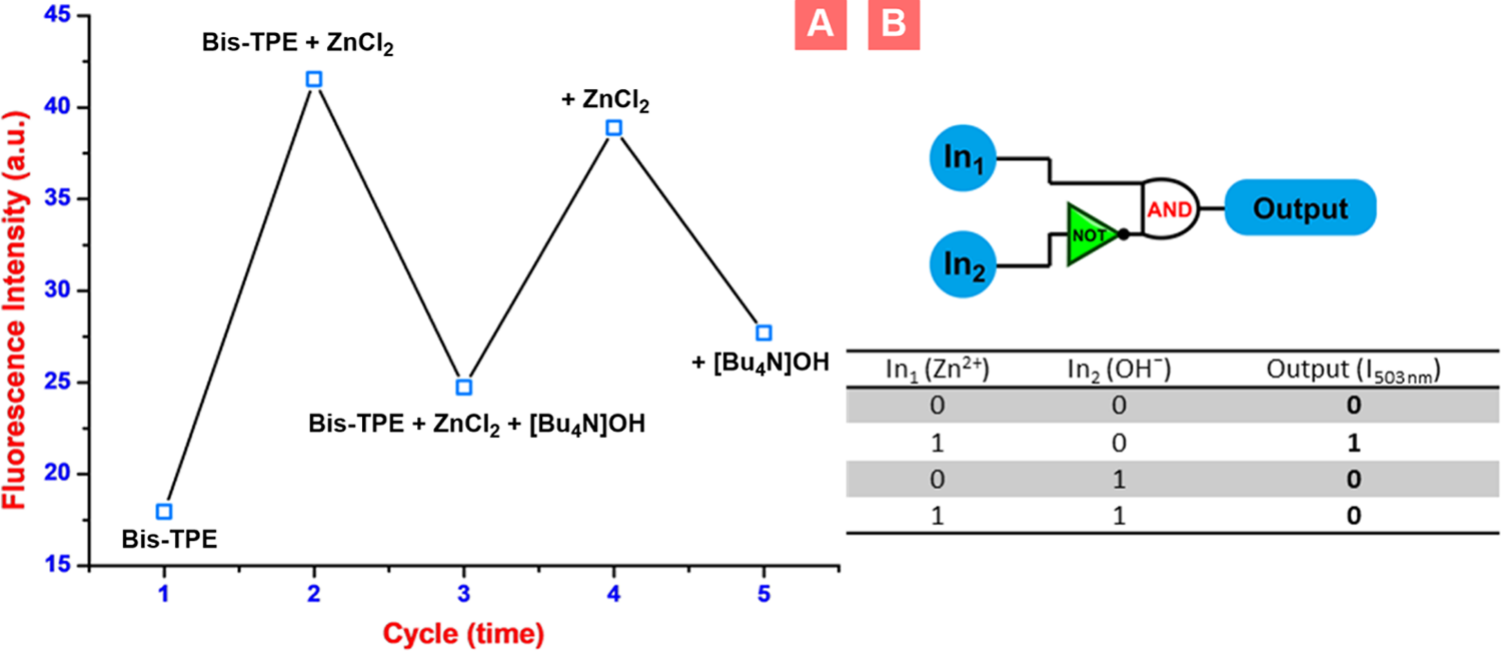
(A) Reversible switching of the fluorescence intensity and (B) the “IMPLICATION” logic gate.

On the other hand, the switchable or reversible sensing properties of chemosensor candidates are another master feature of organic probes. In reversal studies, the alternative addition of ZnCl_2_ and [Bu_4_N]OH to **Bis-TPE** results in switchable on/off variation in the emission intensity at 503 nm (Figures 8A and S13). The studies have shown that **Bis-TPE** could be easily reused for Zn^2+^ and OH^–^ sensing for up to about five cycles (Figure 8A). Moreover, the molecular logic function was built based on the optical behavior of **Bis-TPE** with zinc and hydroxide as inputs. Here, to set up a logic gate, OUTPUT logics 1 and 0 were assigned to turn “on” and “off” fluorescence, respectively. Considering this information, when the data is customized, **Bis-TPE** as a chemosensor remains turned off in the absence of the INPUTS Zn^2+^ (In_1_) and OH^–^ (In_2_). When Zn^2+^ (In_1_) was added to **Bis-TPE**, an increase in emission intensity at 503 nm was noted, resulting in output logic 1, which is a turn-on. On the contrary, when only OH^–^ (In_2_) ions were added to **Bis-TPE**, the decrease observed in the emission peak at 503 nm was noted, resulting in output logic 0 (Figure 8B).

Within the scope of this study, **Bis-TPE** showed a turn-on fluorescence sensor feature against Zn^2+^ ions in the ethanol solvent were determined. The practical feasibility of the existing **Bis-TPE** is compared with some of the previous reports on Zn^2+^ sensors specifically for detection methods and LOD values. When the values in Table 1 are compared, we can say that the values obtained as a result of this study are as acceptable as the values obtained for zinc sensing probes in the literature.^43−47^

**Table 1:** Comparison of Some Zn^2+^ Selective Chemosensors

refs	detection method	sensing ions	LOD (μM)
(39)	fluorescent	Zn^2+^	0.62
(40)	fluorescent	Zn^2+^	0.07
(41)	fluorescent	Zn^2+^	0.10
(42)	fluorescent	Zn^2+^	0.10
(43)	fluorescent	Zn^2+^	0.09
this study	fluorescent	Zn^2+^	0.97

**Figure 9:**
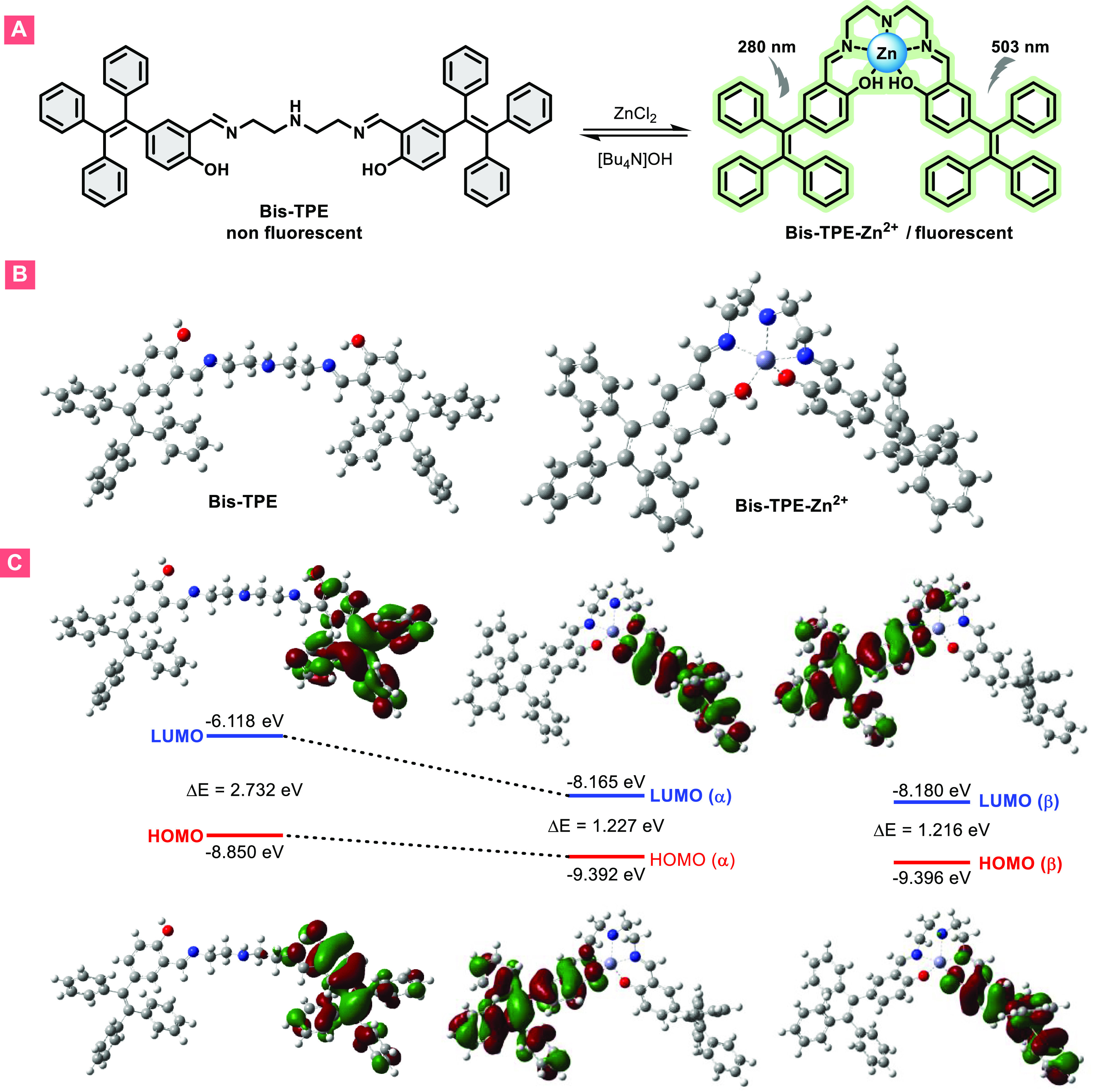
(A) Principle of a “turn-on/off” sensing for Zn^2+^ and OH^–^ detection under UV–vis radiation, (B) ground-state optimized structures of **Bis-TPE** and Bis-TPE-Zn^2+^, and (C) frontier molecular orbitals of **Bis-TPE** in singlet spin and Bis-TPE-Zn^2+^ doublet spin.

Interaction morphology holds significance in sensor studies, and based on the existing literature,^6^ there are three potential binding sites in **Bis-TPE** associated with zinc ions. These sites include the double N (Schiff base) and OH groups of the TPE group and the NH core of the linker (Figure 9A). For this purpose, to understand the interaction mechanism between zinc ions and **Bis-TPE**, ^1^H NMR, and Fourier transform infrared (FT-IR) spectra were utilized (Figures S14B and S15). Initially, a comparison was made between the ^1^H NMR spectra of **Bis-TPE** with and without Zn^2+^ ions (Figure S14A). Accordingly, it can be observed that the proton signals of OH, aliphatic CH_2_, and N=CH experienced some shifts toward the low field. The presence of all functional groups with minor shifts in the FT-IR spectrum also suggests interactions (Figure S15). On the other hand, to gain insight into the structural and electronic properties of **Bis-TPE** and Bis-TPE-Zn^2+^ complexes, density functional theory (DFT) calculations were carried out by using B3LYP in combination with the 6-311G(d,p) basis set for H, C, N, and O atoms and the LANL2DZ basis set for the zinc atom (Gaussian 09).^48^ The corresponding frontier molecular orbitals (MOs) results were obtained from the time-dependent DFT (TD-DFT) computations at the same level. Figure 9B shows the ground-state optimized structures of **Bis-TPE** and Bis-TPE-Zn^2+^. The highest occupied molecular orbital (HOMO) and lowest unoccupied molecular orbital (LUMO) of **Bis-TPE** and Bis-TPE-Zn^2+^ are calculated in the gas phase for the singlet spin and doublet spin, respectively. For compound **Bis-TPE**, the HOMO is delocalized on the TPE center and extends onto the OH–phenyl moieties and the LUMO is mainly localized on the TPE-center moieties with a 2.732 eV band gap. Electron density distribution on Bis-TPE-Zn^2+^ from α-spin HOMO to α-spin LUMO and β-spin HOMO to β-spin LUMO is delocalized on the TPE center and extends onto the OH–phenyl moieties with 1.227 and 1.216 eV band gaps (Figure 9C). Electron density distribution on Bis-TPE-Zn^2+^ from α-spin HOMO – 1 to α-spin LUMO + 1 and β-spin HOMO – 1 to β-spin LUMO + 1 is shown in Figure S16. The band gap reduces from 2.732 to 1.227 and 1.216 eV, which is consistent with the red shift in the absorption spectra.

## Experimental Section

### Synthesis of Bis-TPE

The output molecules (2-(4-methoxyphenyl)ethene-1,1,2-triyl)tribenzene (**3**), 4-(1,2,2-triphenylvinyl)phenol (**4**), and 2-hydroxy-5-(1,2,2-triphenylvinyl)benzaldehyde (**5**) were synthesized following the literature.^43,44^ The detailed experimental procedure and spectroscopic data are given in the Supporting Data file (Figures 2A–C and S1–S3). Following the synthesis of output molecules, the target novel **Bis-TPE** was synthesized as follows. 2-Hydroxy-5-(1,2,2-triphenylvinyl)benzaldehyde (**5**) (500 mg, 1.33 mmol) was dissolved in ethanol (15 mL). To this, a solution of diethylenetriamine (69 mg, 0.66 mmol), also dissolved in the same solvent (5 mL), was added dropwise at room temperature. The reaction mixture was then heated under reflux for 8 h and allowed to cool at room temperature. After the removal of the solvent under reduced pressure, the crude product was purified by recrystallization over ethanol to obtain **Bis-TPE** (500 mg, 92%) as a yellow solid. ^1^H NMR (400 MHz, CDCl_3_) δ 13.27 (bs, 2H), 8.08 (s, 2H), 7.36–6.92 (m, 34H), 6.90 (s, 2H), 6.67 (d, *J* = 8.4 Hz, 2H), 4.01–3.44 (m, 4H), 3.10–2.58 (m, 4H). ^13^C NMR (100 MHz, CDCl_3_) δ 166.4, 166.3, 160.2, 144.0, 143.9, 140.6, 140.1, 135.9, 134.5, 134.4, 131.7, 131.6, 128.1, 128.0, 127.9, 126.8, 126.7, 126.6, 118.28, 118.27, 116.6, 59.5, 49.9 (Figure S4).

### Procedures of Measurement of Photophysical Properties

The UV–vis and fluorescence studies of **Bis-TPE** with various ions were recorded following the introduction of the ions (1 equiv) at room temperature each time in ethanol. The fluorescence titration study of **Bis-TPE** with ZnCl_2_ was recorded by adding corresponding concentrations of ZnCl_2_ to a solution of **Bis-TPE** in EtOH. Each measurement was repeated at least twice until consistent values were obtained. In addition, the Job’s plot measurement was performed, and following this, the LOD, LOQ, and *K*_a_ values were calculated with related formulas. The detailed experimental procedures are given in the Supporting Data.

## Conclusions

In conclusion, novel **Bis-TPE** was synthesized and its wide range of photochemical properties was investigated. For this purpose, the interactions of **Bis-TPE** with a wide range of ions were studied in a variety of solvent systems, and **Bis-TPE** showed turn-on sensor features against Zn^2+^ in ethanol. Moreover, the Bis-TPE-Zn^2+^ system showed specific turn-off fluorescence features against hydroxide ions. This also means reversible sensing properties and reusable **Bis-TPE** after the interaction with zinc. The LOD, LOQ, and *K*_a_ values of Bis-TPE-Zn^2+^ were calculated as 0.97 (970 nm) μM, 2.96 μM, and 3.76 × 10^5^ M^–1^, respectively. Ultimately, effective processes were established for the synthesis of **Bis-TPE** and the detection of zinc and hydroxide in ethanol.

## Supporting Information Available

General experimental methods and characterizations of the materials (PDF)
