# Supplementary material for: Synthesis of Bis-tetraphenylethene as a Novel Turn-On Selective Zinc Sensor
Source: ACS Omega. 2023 Jul 3;8(28):25432–40. doi: 10.1021/acsomega.3c02955 (PMC10357583; doi:10.1021/acsomega.3c02955)
Supplement: Supplementary file 1 — ao3c02955_si_001.pdf [file ao3c02955_si_001.pdf]

<u>**Supplementary Data for**</u>

Synthesis of bis-tetraphenylethene as a novel turn-on selective zinc sensor

Abdullah Saleh Hussein[a,b], Ferruh Lafzi*[c], Haydar Kilic[c] and Sinan Bayindir*[a]

[a]*Department of Chemistry, Faculty of Sciences and Arts, Bingöl University, Bingöl, Türkiye*
[b]*College of Education Chemistry Department, Salahaddin University-Erbil, Erbil, Iraq*
[c]*Department of Chemistry, Faculty of Sciences, Atatürk University, Erzurum, Türkiye*

*Corresponding author:

E-mail address: farrokh.lafzi@atauni.edu.tr (F. Lafzi)

E-mail address: sbayindir@bingol.edu.tr (S. Bayindir)

General methods

All chemicals, reagents, and solvents were commercially available from Sigma-Aldrich or Merck. Infrared spectra were recorded on a Mattson 1000 FT-IR spectrophotometer. ^{1}H NMR and ^{13}C NMR spectra were recorded on a 400 (100)-MHz Varian and Bruker spectrometer and are reported in terms of chemical shift (δ, ppm) with $SiMe_4$ as an internal standard. Data for ^{1}H NMR are recorded as follows: chemical shift (δ, ppm), multiplicity (s: singlet, d: doublet, t: triplet, q: quartet, p: pentet, m: multiplet, bs: broad singlet, bd: broad doublet, qd: quasi doublet) and coupling constant (s) in Hz, integration. Elemental analyses were carried out on a LECO CHNS-932 instrument. Column chromatography was carried out on silica gel 60 (230–400 mesh ASTM). The reaction progress was monitored by thin-layer chromatography (TLC) (0.25-mm-thick precoated silica plates: Merck Fertigplatten Kieselgel (60 F254)). UV-Vis absorption and fluorescence spectra of samples were recorded on a Shimadzu UV-3101PL UV-Vis-NIR spectrometer and Perkin–Elmer (Model LS 55) Fluorescence Spectrophotometer, respectively.

The synthesis of (2-(4-methoxyphenyl)ethene-1,1,2-triyl)tribenzene (3)[1]. To a solution of diphenyl methane (2.5 g, 14.9 mmol) in anhydrous THF (30 mL) *n*-butyllithium (2.5 M in hexane, 5.9 mL, 14.9 mmol) was added at 0 ^{o}C under nitrogen. After stirring for 2 h at the same temperature 4-methoxybenzophenone (3.15 g, 14.9 mmol) in THF (10 mL) was added to the the reaction mixture and stirred for 12 h to allow the temperature rise gradually to room temperature. Then the reaction mixture was quenched by adding an aqueous solution of ammonium chloride and then the mixture was extracted with dichloromethane (3 × 50 mL). The organic layers were combined and dried over anhydrous Na_2SO_4, and the solvent was evaporated to give a crude alcohol intermediate. The resulting alcohol intermediate was dissolved in toluene (60 mL), and *p*-toluenesulfonic acid (PTSA, 500 mg) was added and refluxed for 16 h. After the mixture was cooled to room temperature was evaporated on a rotary evaporator to give a crude residue which was purified by silica gel chromatography using DCM/PE (petroleum ether) (v/v = 1/10) as an eluent to yield compound **3** (4.7 g, 87%) as a white powder. ^{1}H NMR (400 MHz, $CDCl_3$) δ 7.36 – 7.03 (m, 15H), 7.02 – 6.84 (m, AA′ part of AA′BB′ system, 2H), 6.80 – 6.59 (m, BB′ part of AA′BB′ system, 2H), 3.77 (s, 3H). ^{13}C NMR (100 MHz, $CDCl_3$) δ 158.1, 144.1, 144.0, 140.5, 140.1, 136.1, 132.5, 131.4, 131.38, 131.35, 127.7, 127.6, 126.4, 126.3, 113.1, 55.1 (3C signals overlaps).

The synthesis of 4-(1,2,2-triphenylvinyl)phenol (4) [1]. To a solution of (2-(4-methoxyphenyl)ethene-1,1,2-triyl)tribenzene (**3**) (2.0 g, 5.52 mmol) in $CHCl_3$ (30 mL) at -78 ^{o}C, a solution of BBr_3 (0.69 mL, 5.52 mmol) in $CHCl_3$ (5 mL) was added slowly with dropping funnel under stirring. After addition, the mixture was warmed to room temperature and stirred for 12 h. The reaction was carefully quenched with NH_4Cl solution using an ice bath and the resulting solution was washed with water (2×30 mL), dried over Na_2SO_4, and evaporated. The crude product was purified by column chromatography on silica gel using ethyl

acetate (EA)/PE (v/v = 1/10) to give compound **4** as a white solid (1.77 g, 92%). ^{1}H NMR (400 MHz, $CDCl_3$) δ 7.24 – 6.99 (m, 15H), 6.98 – 6.75 (m, AA′ part of AA′BB′ system, 2H), 6.74 – 6.28 (m, BB′ part of AA′BB′ system, 2H), 4.64 (s, 3H). ^{13}C NMR (100 MHz, CDCl3) δ 154.0, 144.0, 143.9, 143.89, 140.4, 140.2, 136.4, 132.8, 131.38, 131.35, 131.3, 127.7, 127.6, 126.4, 126.3, 114.6 (2C signals overlaps).

The synthesis of 2-hydroxy-5-(1,2,2-triphenylvinyl)benzaldehyde (5)[2]. The 4-(1,2,2-triphenylvinyl) phenol (**4**) (1.0 g, 2.87 mmol) was added under vigorous stirring to a solution of hexamethylenetetramine (2.01 g, 14.35 mmol) in trifluoroacetic acid (15 mL), which was refluxed 4 h, then quenched with water (30 mL) and extracted with CH_2Cl_2 (3 × 20 mL). The combined organic layers were washed with saturated Na_2CO_3 (20 mL), water and brine, then dried over anhydrous Na_2SO_4. The solvent was removed under reduced pressure and the residue was purified by column chromatography to give **5** as a yellow solid (0.71 g, 65%). ^{1}H NMR (400 MHz, $CDCl_3$) δ 10.84 (s, 1H), 9.50 (s, 1H), 7.15 – 7.09 (m, 2H), 7.08 – 7.00 (m, 9H), 7.00 – 6.91 (m, 6H), 6.64 (d, *J* = 9.3 Hz, 1H). ^{13}C NMR (100 MHz, $CDCl_3$) δ 196.5, 160.2, 143.4, 143.3, 143.0, 141.5, 140.1, 138.9, 136.4, 135.6, 131.34, 131.3, 131.2, 128.0, 127.9, 127.7, 126.83, 126.8, 126.6, 120.2, 117.0.

UV-Vis and fluorescence studies of Bis-TPE with various cations and anions: The solution of **Bis-TPE** (1×10^{-2} M) and cations (Al^{3+}, Ca^{2+}, Cd^{2+}, Co^{2+}, Cu^{2+}, Fe^{2+}, Fe^{3+}, Hg^{2+}, Mg^{2+}, Mn^{2+}, Ni^{2+}, Zn^{2+}, and Pb^{2+} as their chloride salts, 1×10^{-2} M)/anions (([Bu_4N]F, [Bu_4N]Cl, [Bu_4N]Br, [Bu_4N]I, [Bu_4N]AcO, [Bu_4N]BnO, [Bu_4N]HSO_4, [Bu_4N]ClO_4, [Bu_4N]CN, [Bu_4N]SCN, [Bu_4N]H_2PO_4, [Bu_4N]OH, 1×10^{-2} M) were prepared in CH_3CN and H_2O, respectively. A solution of **Bis-TPE** (10 μM) was placed in a quartz cell and the UV-Vis and fluorescence spectrums were recorded in EtOH. After introduction of the solution of cations (1 equiv.), the changes in absorbance intensity were recorded at room temperature each time.

The fluorescence titration of Bis-TPE with ions: The solution of probe **Bis-TPE** (1×10^{-2} M) and ions (1×10^{-2} M) were prepared in EtOH and H_2O, respectively. The concentration of probe **Bis-TPE** used in the experiments was 10 μM. The fluorescence titration spectra were recorded by adding corresponding concentration of ions to a solution of BİS-TPE in EtOH or H_2O. Each titration was repeated at least twice until consistent values were obtained.

Job's plot measurement: Probe **Bis-TPE** was dissolved in EtOH to make the concentration of 1×10^{-2} M. 5.00, 4.50, 4.00, 3.50, 3.00, 2.50, 2.00, 1.50, 1.00, 0.50 and 0.0 mL of the ligand solution were taken and transferred to vials. Ions were dissolved in H_2O to make the concentration of 1×10^{-2} M. 0.0, 0.50, 1.00, 1.50, 2.00, 2.50, 3.00, 3.50, 4.00, 4.50, and 5 mL of the ions solution were added to each ligand solution.

Each vial had a total volume of 5 mL. After shaking the vials for a few seconds, fluorescence spectra were taken at room temperature.

Determination of detection limit: The absorption and fluorescence measurements were taken for each solution containing Zn^{2+} ions. The detection limits for ions were calculated based on the absorption and fluorescence titration. For this purpose, $3\,s/k$ equation was used. Where s the standard deviation of blank, k is the slope of the fit line in fluorescence titration experiment or in absorbance titration experiment.

LOQ = 3.3 x (Standard Deviation (SD) / Slope) (1)

LOD = 10 x (Standard Deviation (SD) / Slope) (2)

Determination of Association Constant: Association constant was calculated according to the Benesi-Hildebrand equation by fluorescence method. Association Constant (K_a) was calculated following the equation stated below:

$$\frac{1}{F-F_0} = \frac{1}{\{K_a(F_{max}-F_0)[M^{x+}]^n\}} + \frac{1}{F_{max}-F_0}$$

Here, F_0 is the fluorescence of receptor in the absence of metal ion, F is the fluorescence recorded in the presence of added metal ion, F_{max} is fluorescence in presence of added $[M^{x+}]$ max and K_a is the association constant, where $[M^{x+}]$ is ions n is the binding stoichiometry for receptor and ions. The association constant (K_a) could be determined from the slope of the straight line of the plot of $1/F\text{-}F_o$ against $1/[M]^{1/2}$.

The pH measurement: The effect of different pH environments (range of 2–12) was studied for the practical application of the probe **Bis-TPE** (10 µM, in EtOH) in the absence and presence of Zn^{2+} (30 µM, in H_2O). For this purpose, the **Bis-TPE** samples were prepared in ten different tubes, and zinc ions dissolved in water were added. The pH values of samples were modulated by adding HCl or NaOH solution. The pH values of the solution were monitored with a pH meter and/or pH stick.

FTIR studies: The FTIR peaks around 3384 cm^{-1} and 3318 cm^{-1} can be due to the symmetric and asymmetric stretching frequency of the O-H and –N–H (NH or NH_2) bond in the primary or secondary amino group. Additionally, one band, due to in-plane =C-H bending of the TPE core, seems to appear in the region around 1023 cm^{-1} and 1025 cm^{-1} (Figure S14).

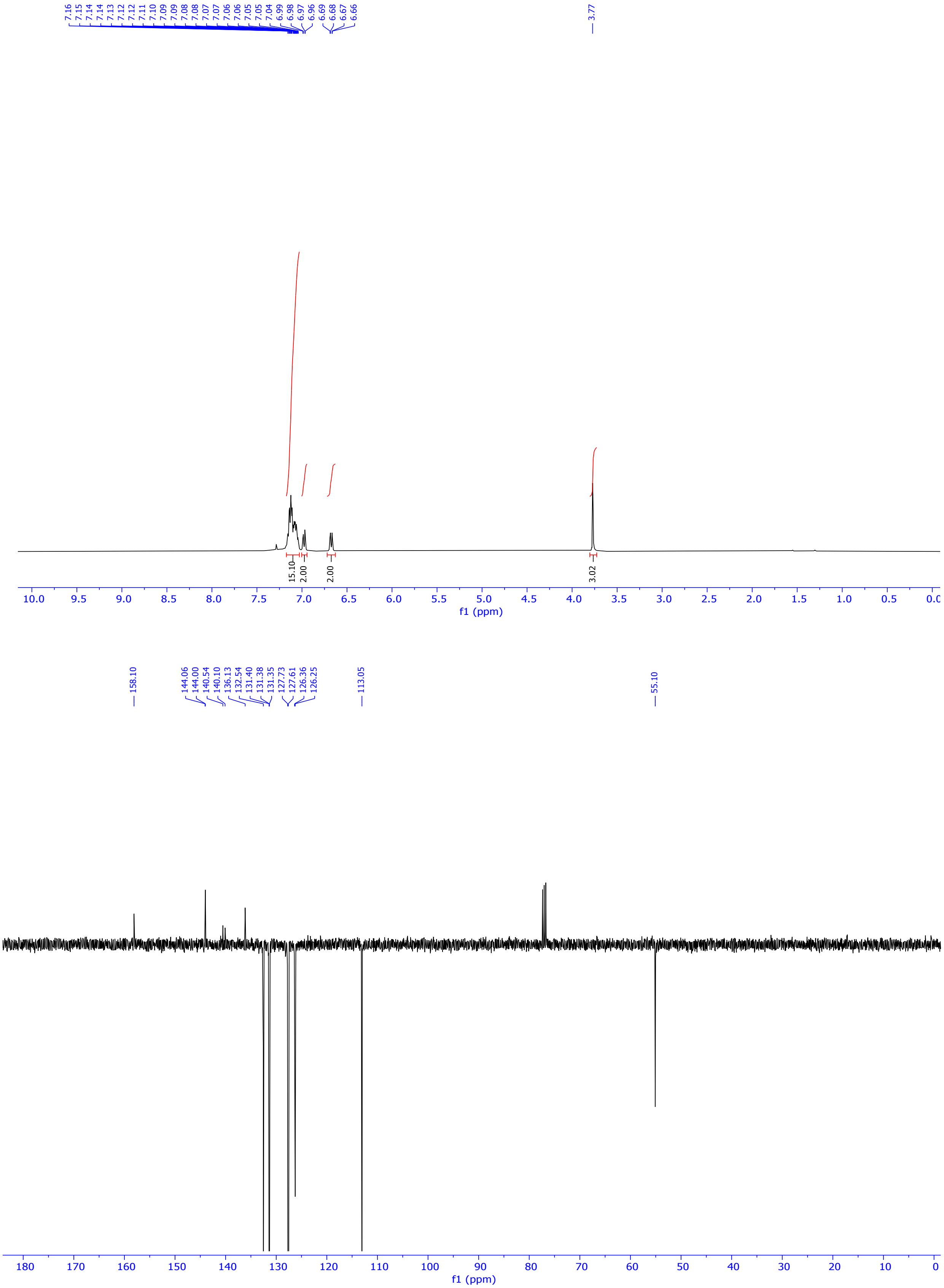

Figure S1. The ^{1}H-NMR (400 MHz) and APT ^{13}C-NMR (100 MHz) spectrums of (2-(4-methoxyphenyl)ethene-1,1,2-triyl)tribenzene (**3**) in $CDCl_3$.

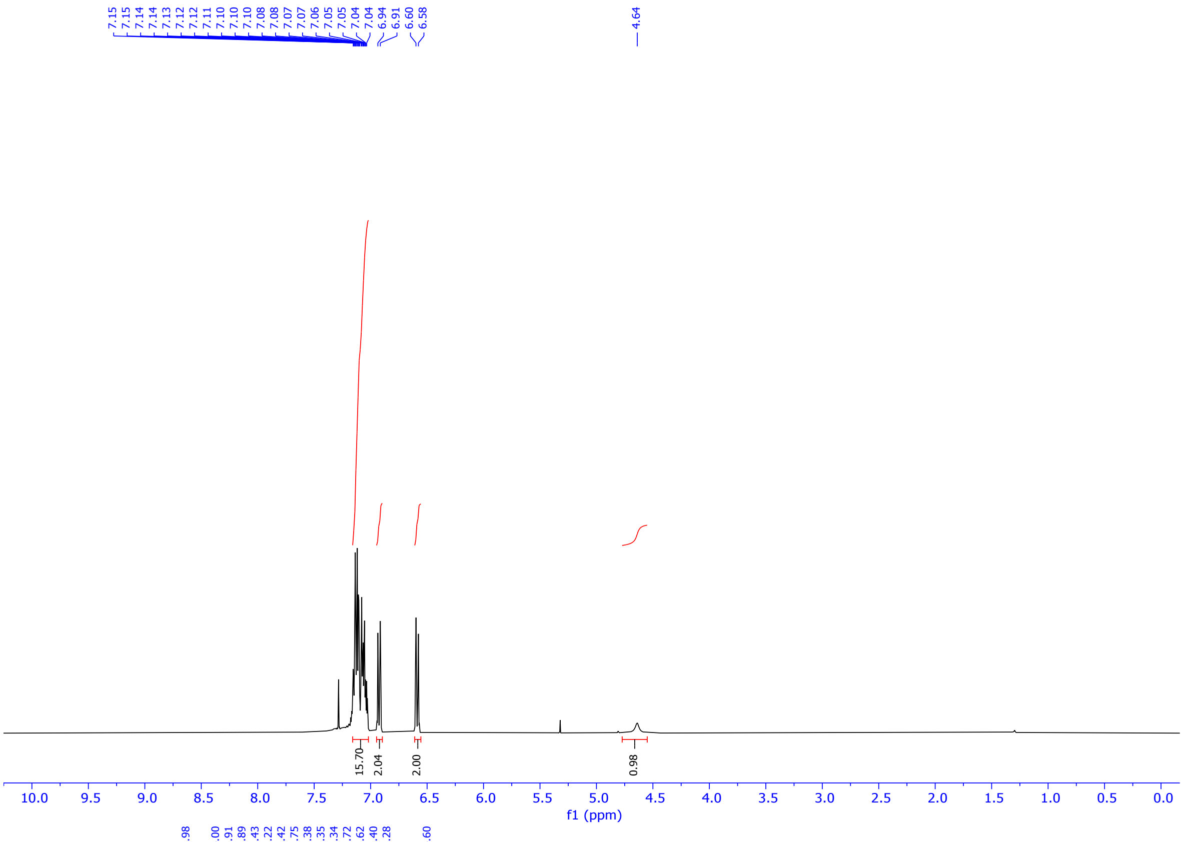

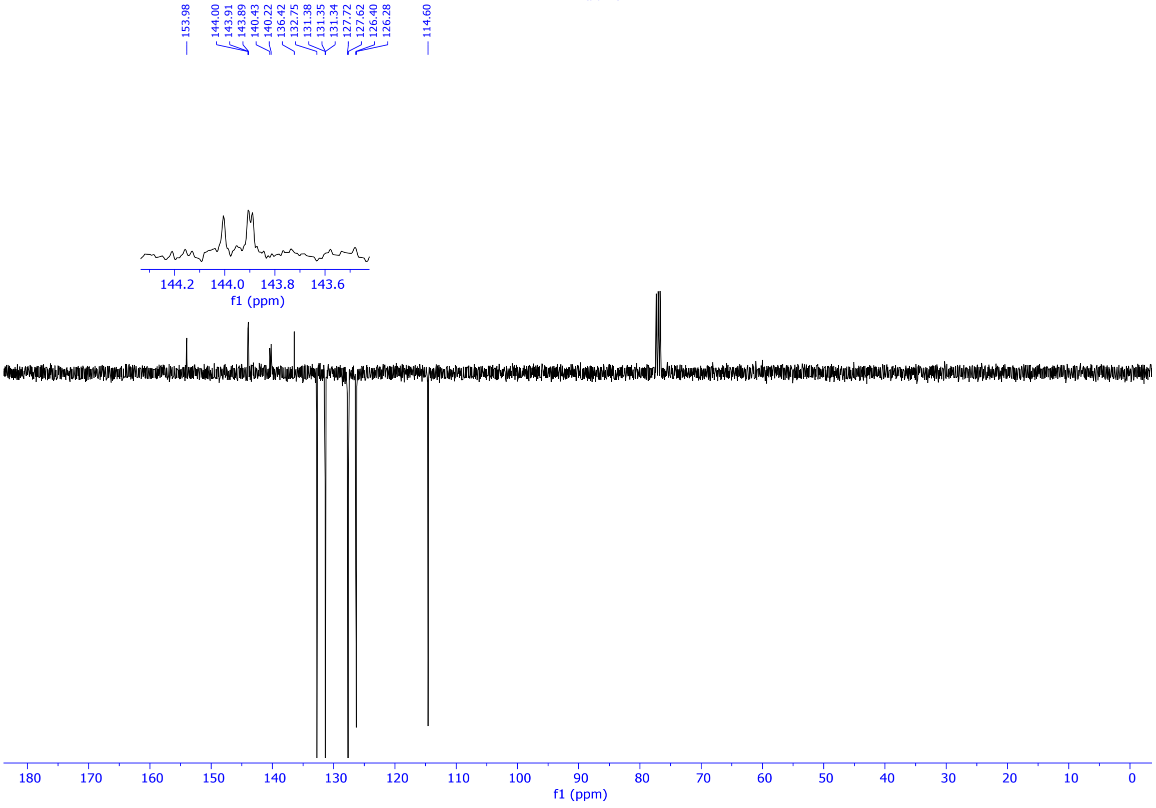

Figure S2. The ^{1}H-NMR (400 MHz) and APT ^{13}C-NMR (100 MHz) spectrums of 4-(1,2,2-triphenylvinyl)phenol (**4**) in $CDCl_3$.

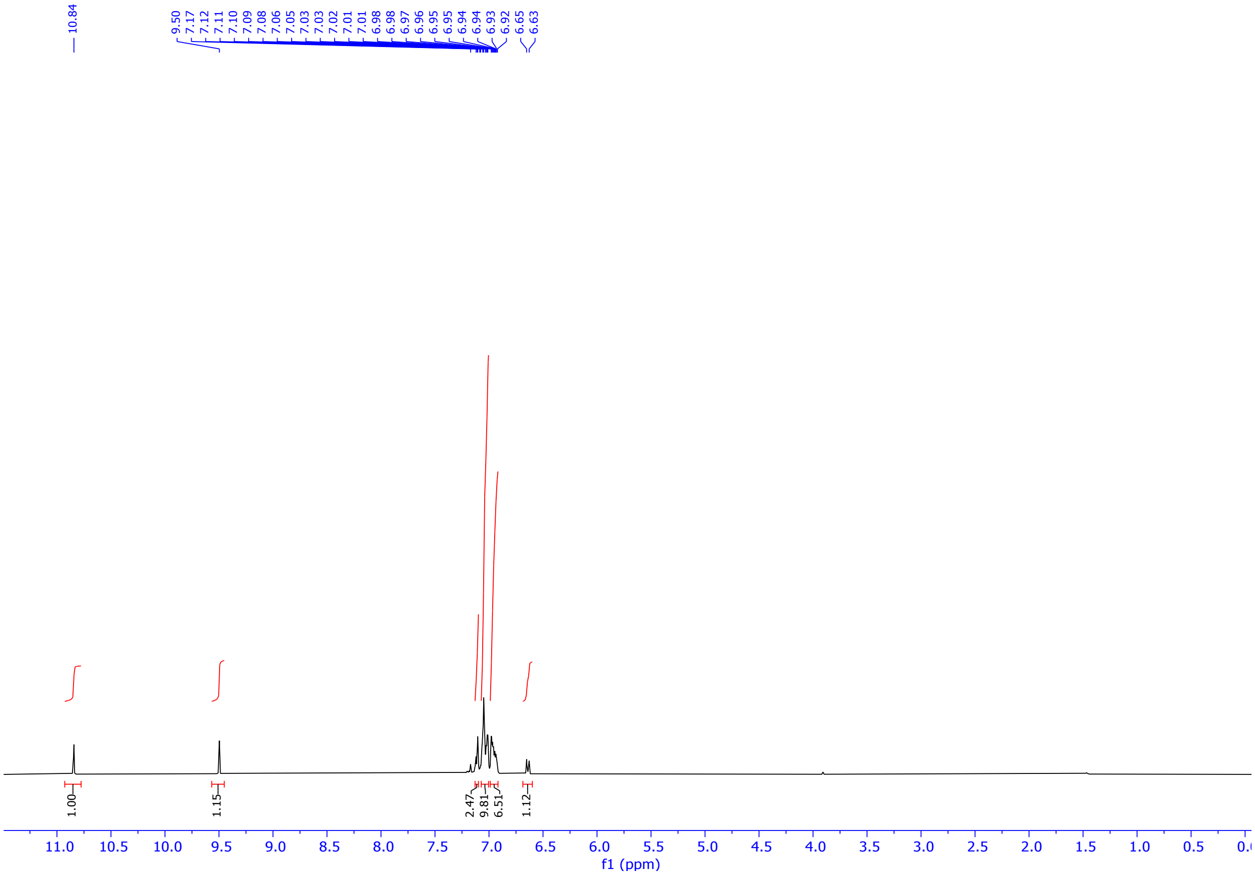

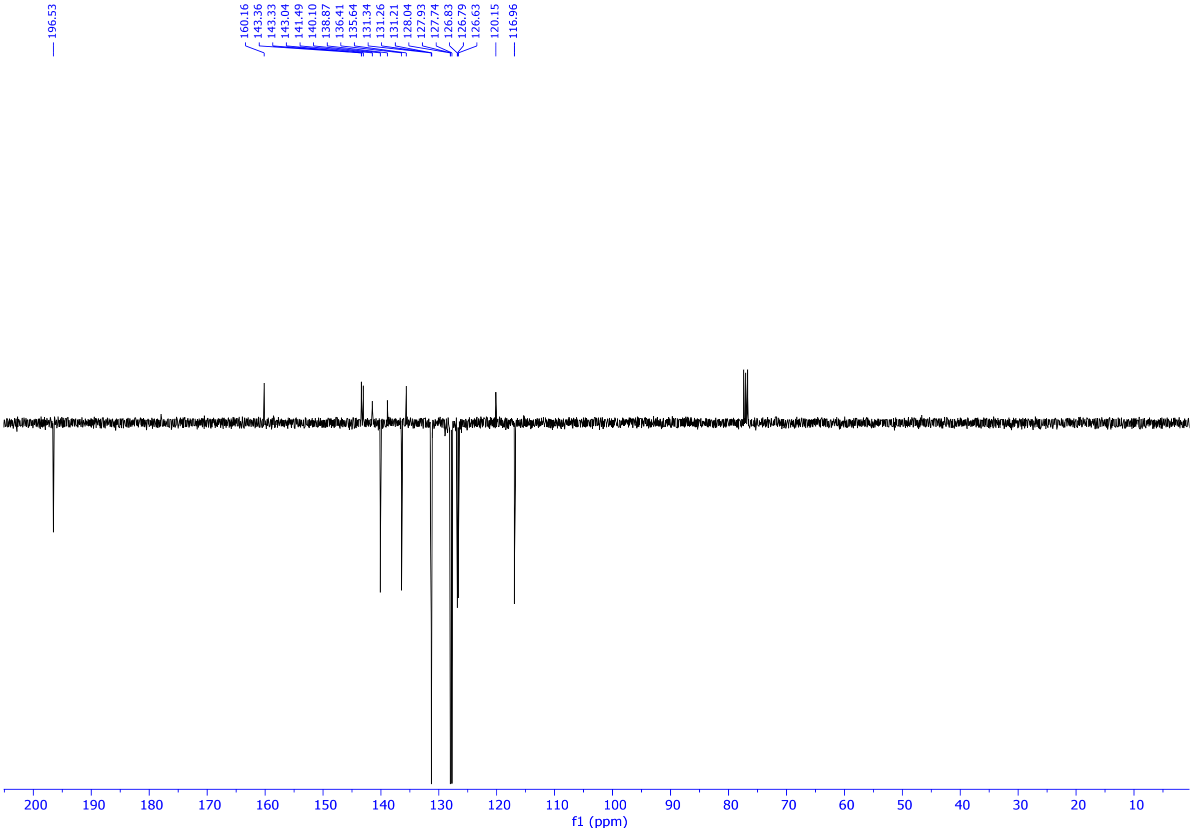

Figure S3. The ^{1}H-NMR (400 MHz) and APT ^{13}C-NMR (100 MHz) spectrums of 2-hydroxy-5-(1,2,2-triphenylvinyl)benzaldehyde (**5**) in $CDCl_3$.

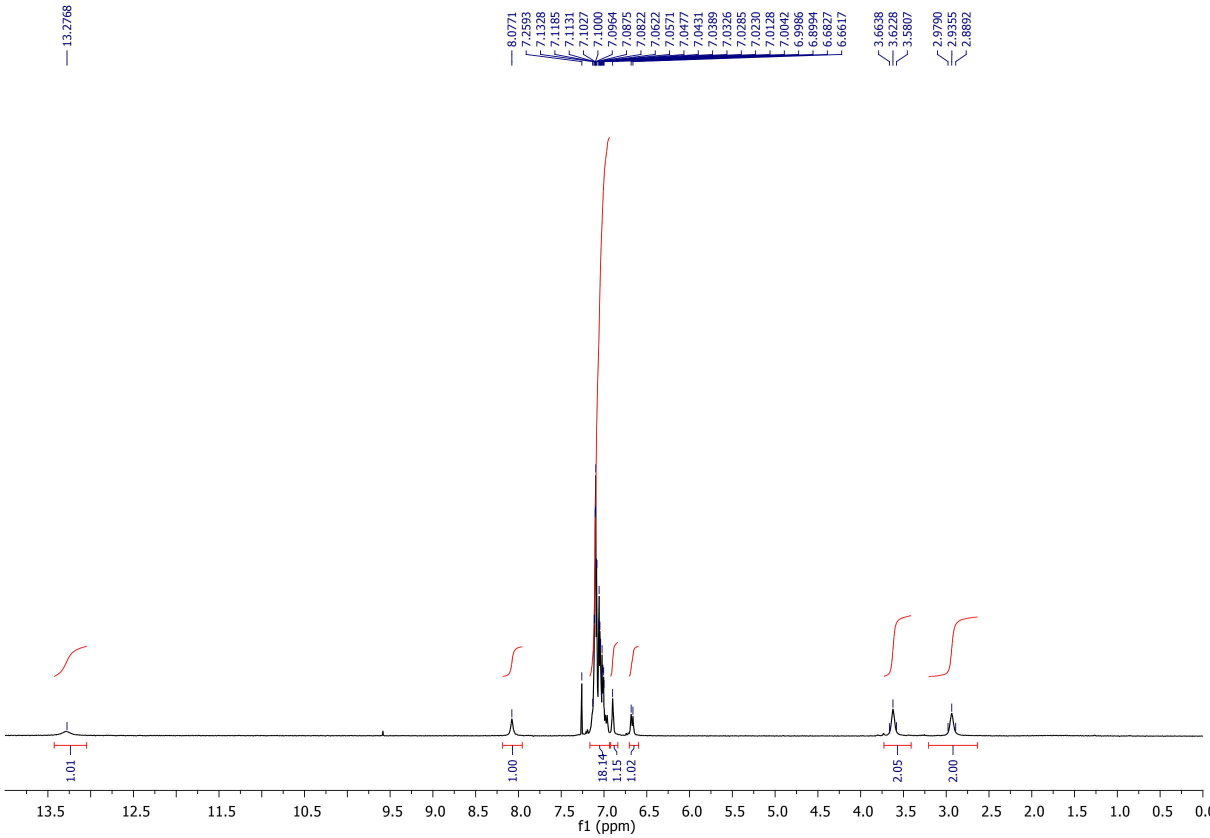

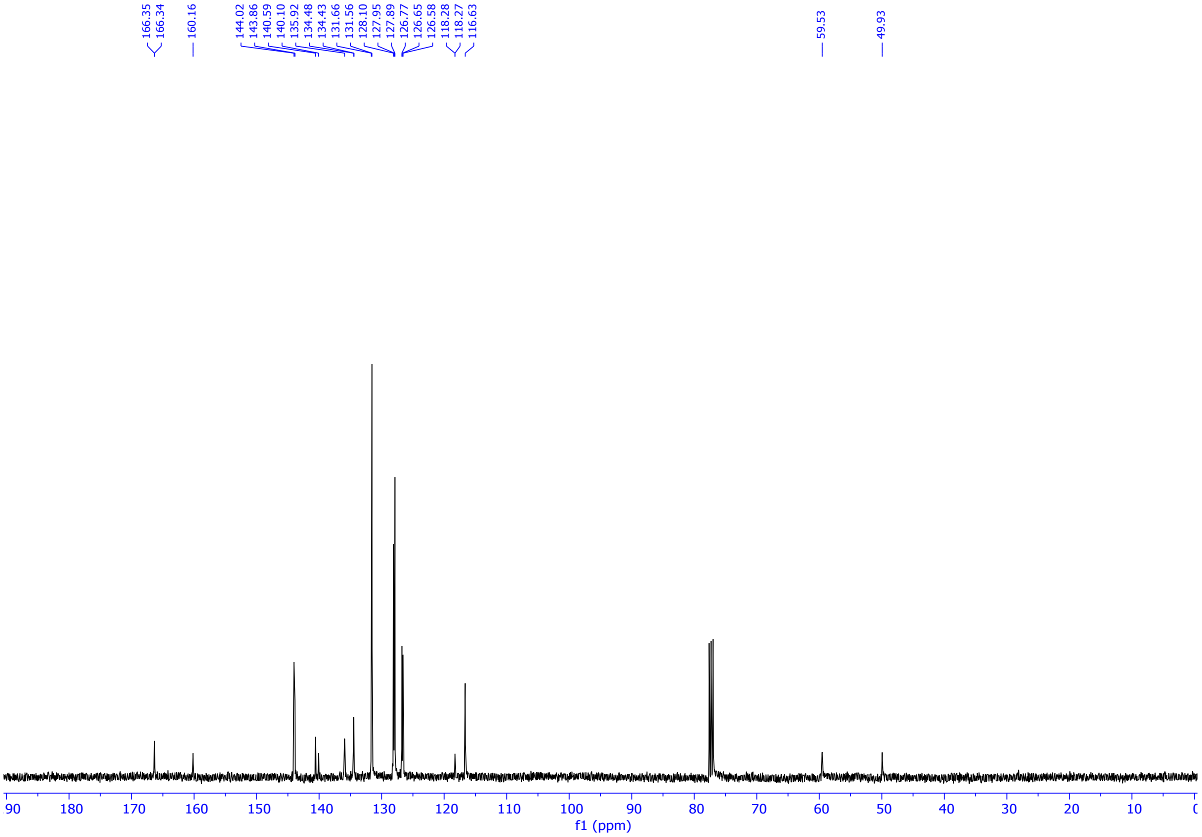

Figure S4. The ^{1}H-NMR (400 MHz) and APT ^{13}C-NMR (100 MHz) spectrums of Bis-TPE in $CDCl_3$.

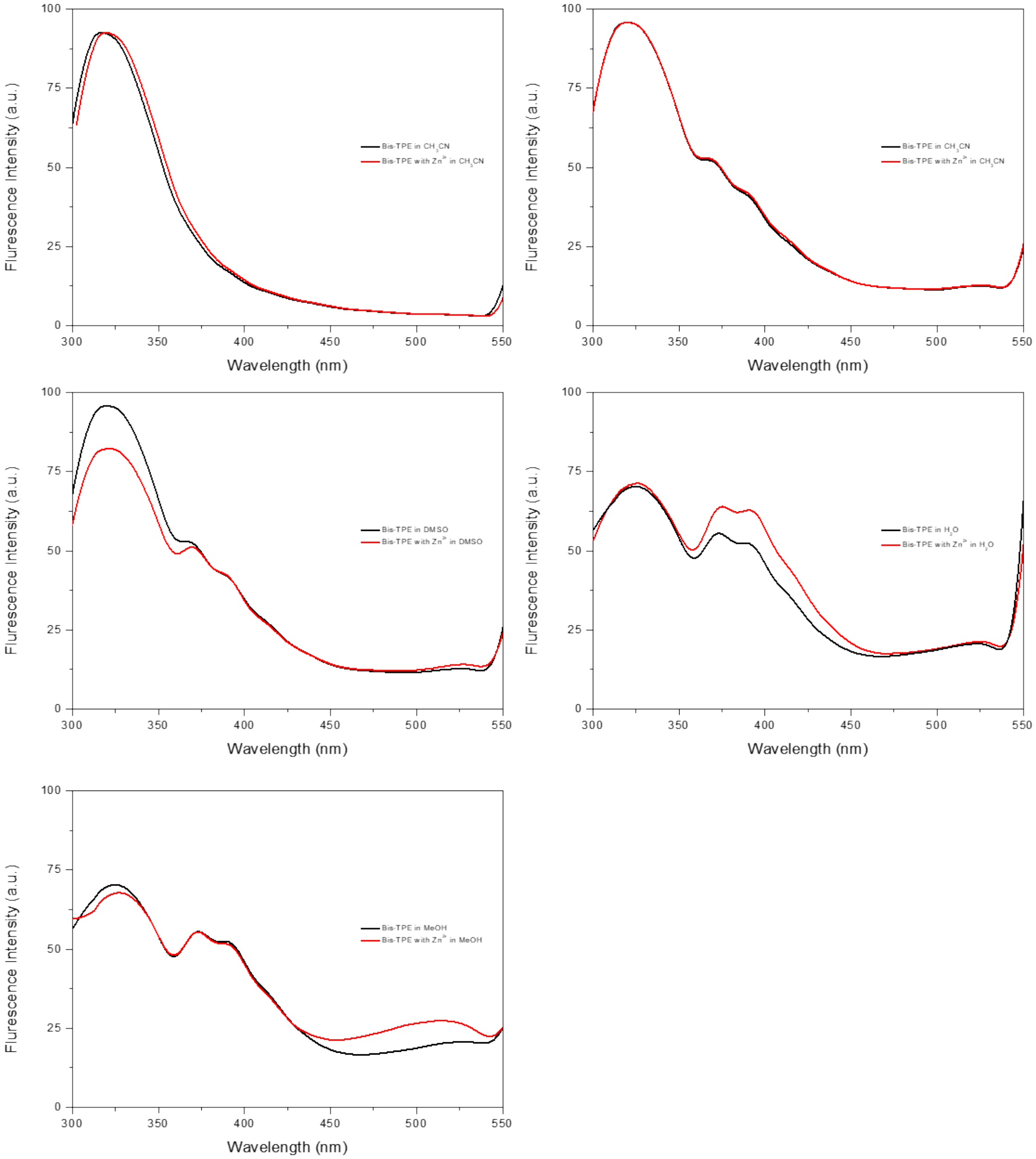

Figure S5. Fluorescence spectra of Bis-TPE and Bis-TP-Zn^{2+} in variety solvent systems.

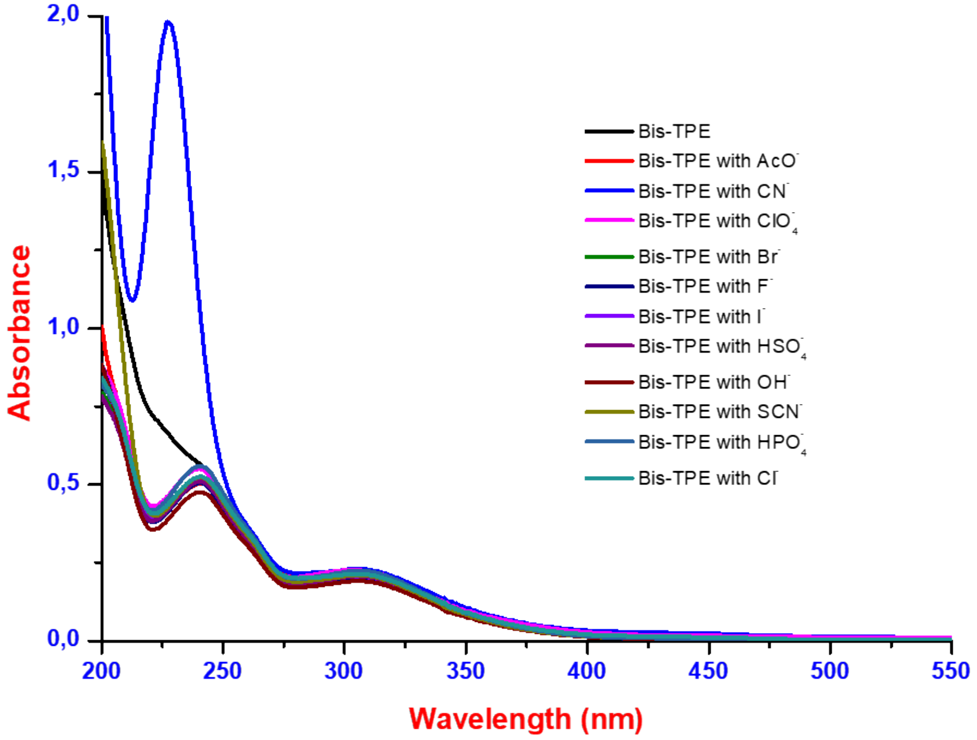

Figure S6. Absorbance spectra of Bis-TPE absence and presence of metal ions in EtOH

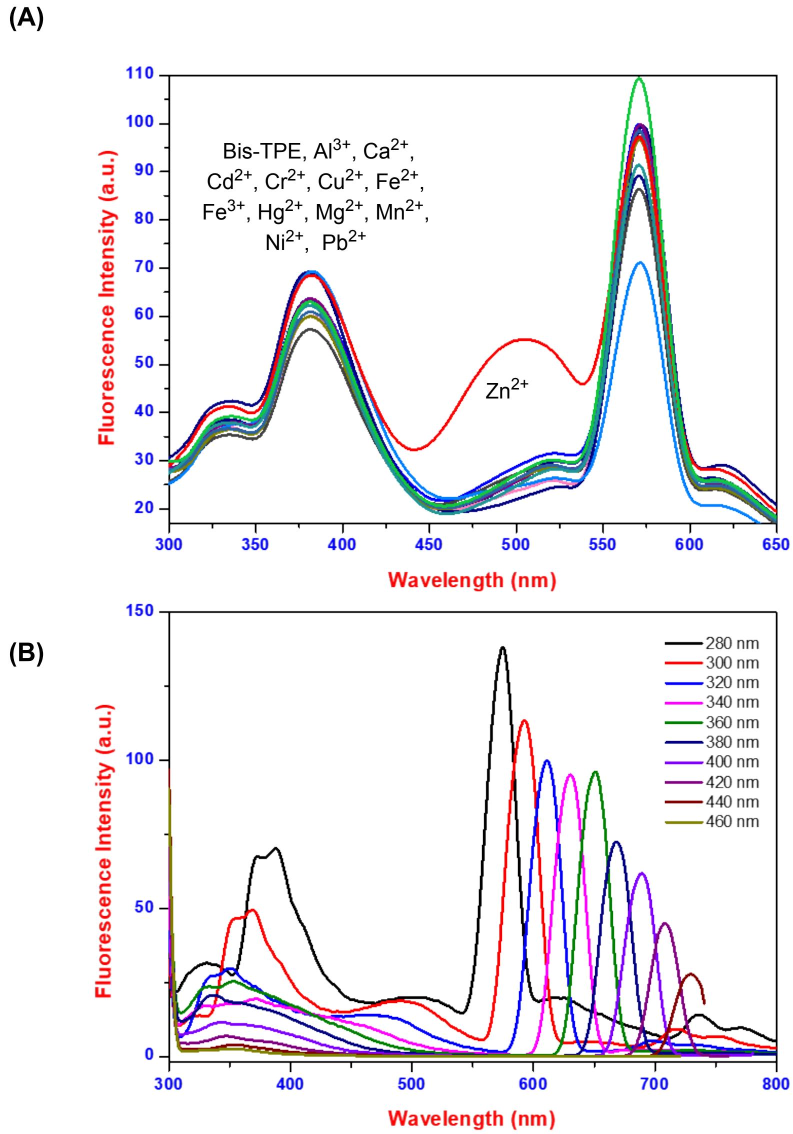

Figure S7. (**A**) Fluorescence spectra of Bis-TPE in the presence of metal ions in EtOH, and (**B**) the excitation wavelength dependency studies of Bis-TPE.

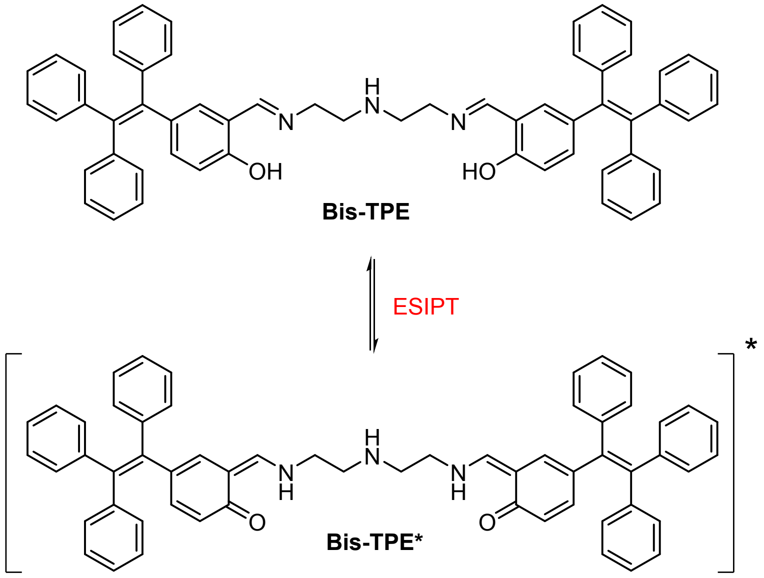

Scheme S1. The proposed mechanism for the process of ESIPT of Bis-TPE.

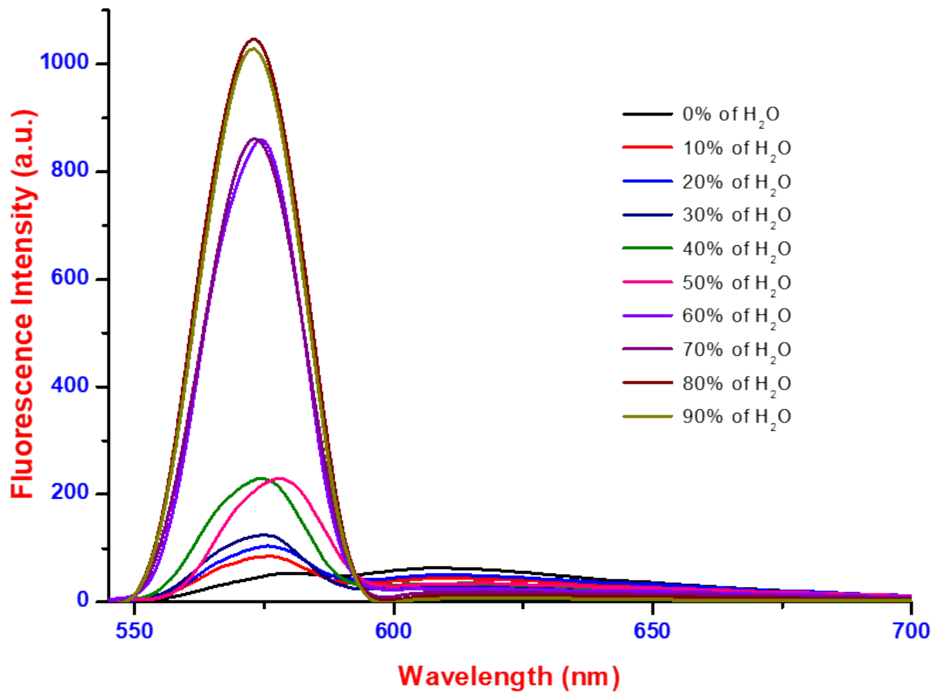

Figure S8. The AIE fluorescent response of Bis-TPE in different water ratio mixtures

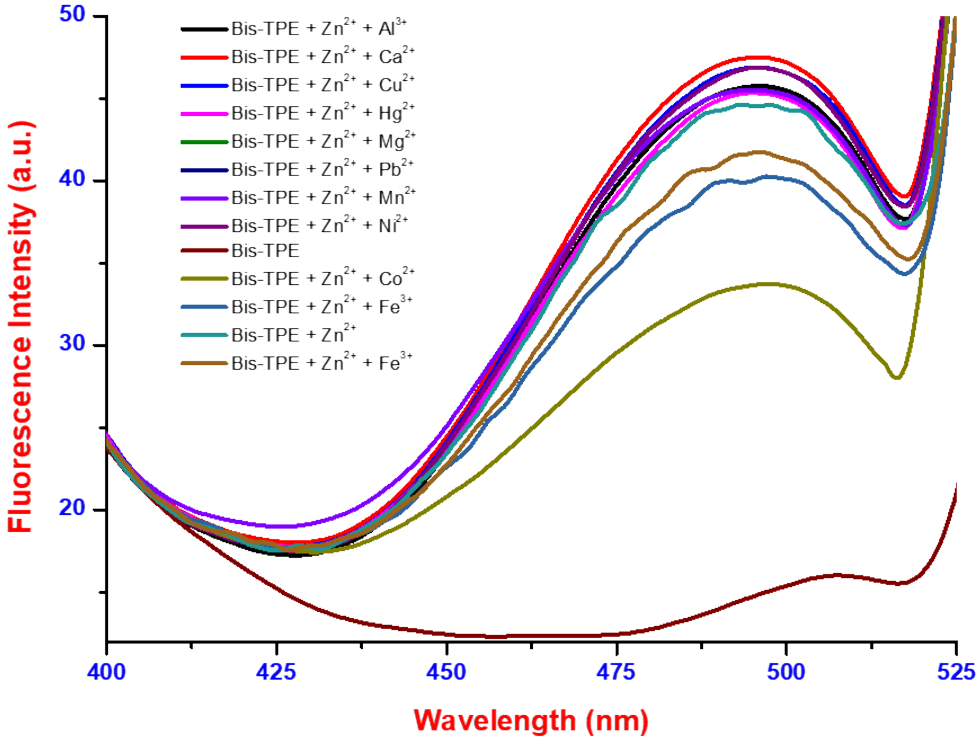

Figure S9. Fluorescence spectra of Bis-TPE in the presence of Zn^{2+} upon the addition of different metal ions in EtOH to 1 equiv.

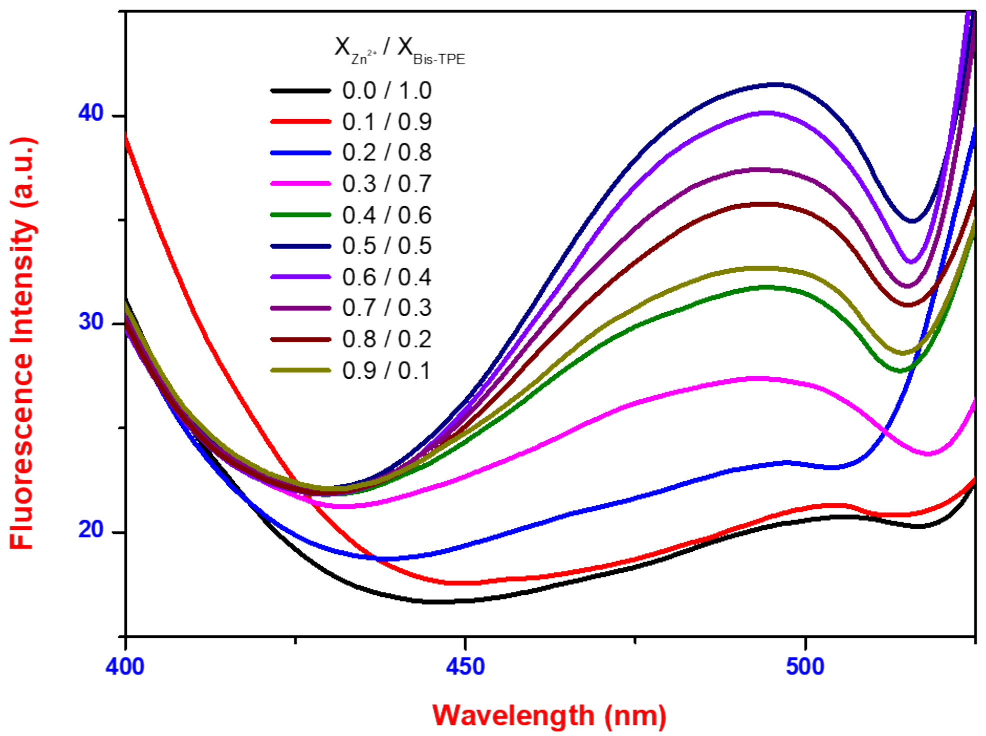

Figure S10. The Job's plot fluorescence spectra of Bis-TPE with Zn^{2+} in EtOH

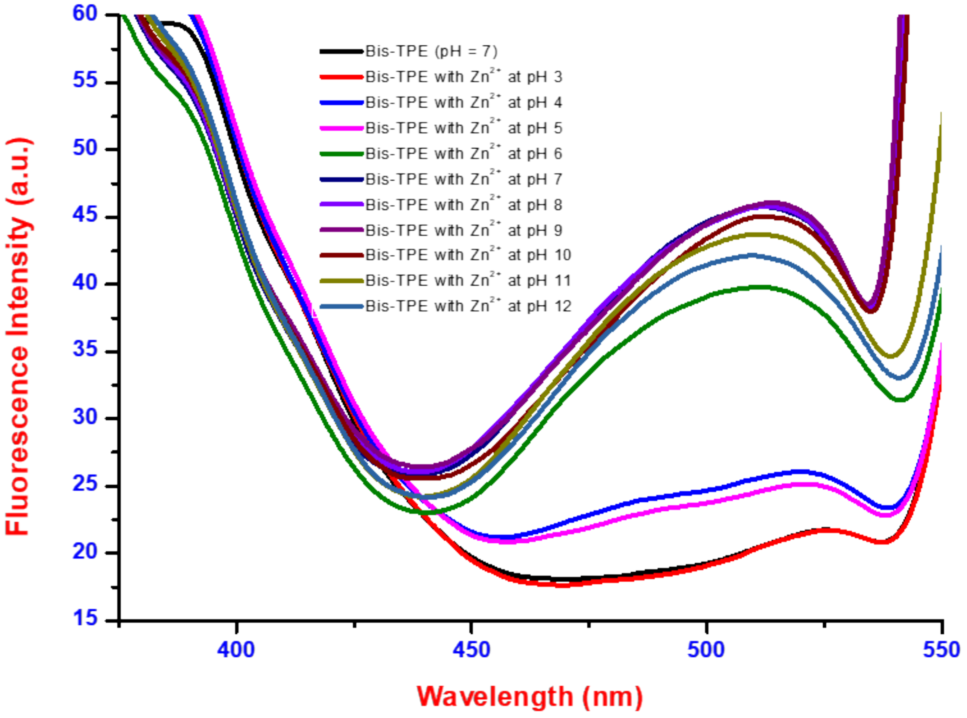

Figure S11. The fluorescence spectra of Bis-TPE with [$ZnCl_2$] at different pH (3–11) in EtOH, the pH was modulated by adding HCl or NaOH solution.

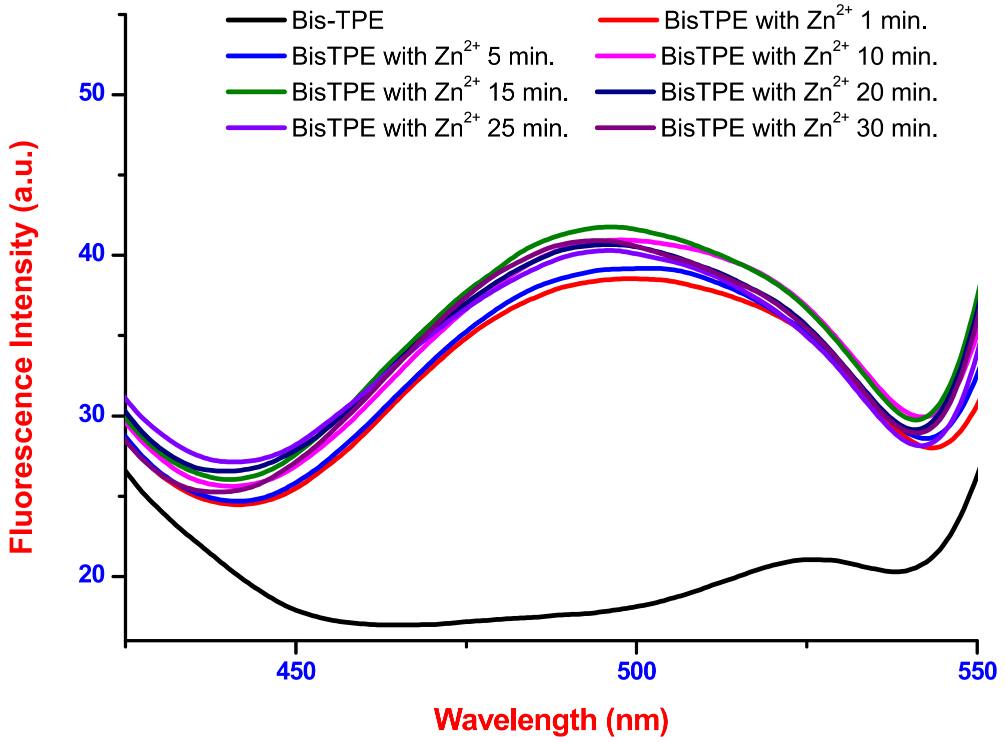

Figure S12. The fluorescence enhancing profile of addition Zn^{2+} to Bis-TPE in EtOH from 0 min to 30 min.

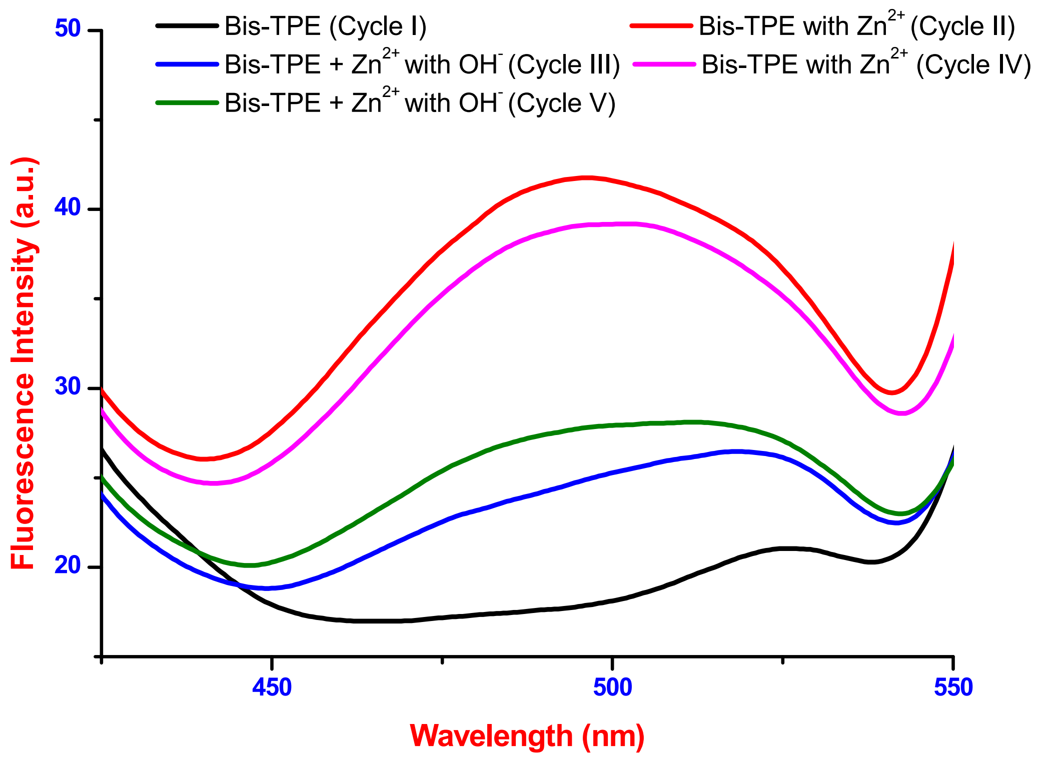

Figure S13. Reversible switching of the fluorescence spectrum of Bis-TPE upon alternate addition of Zn^{2+} and OH^{-}.

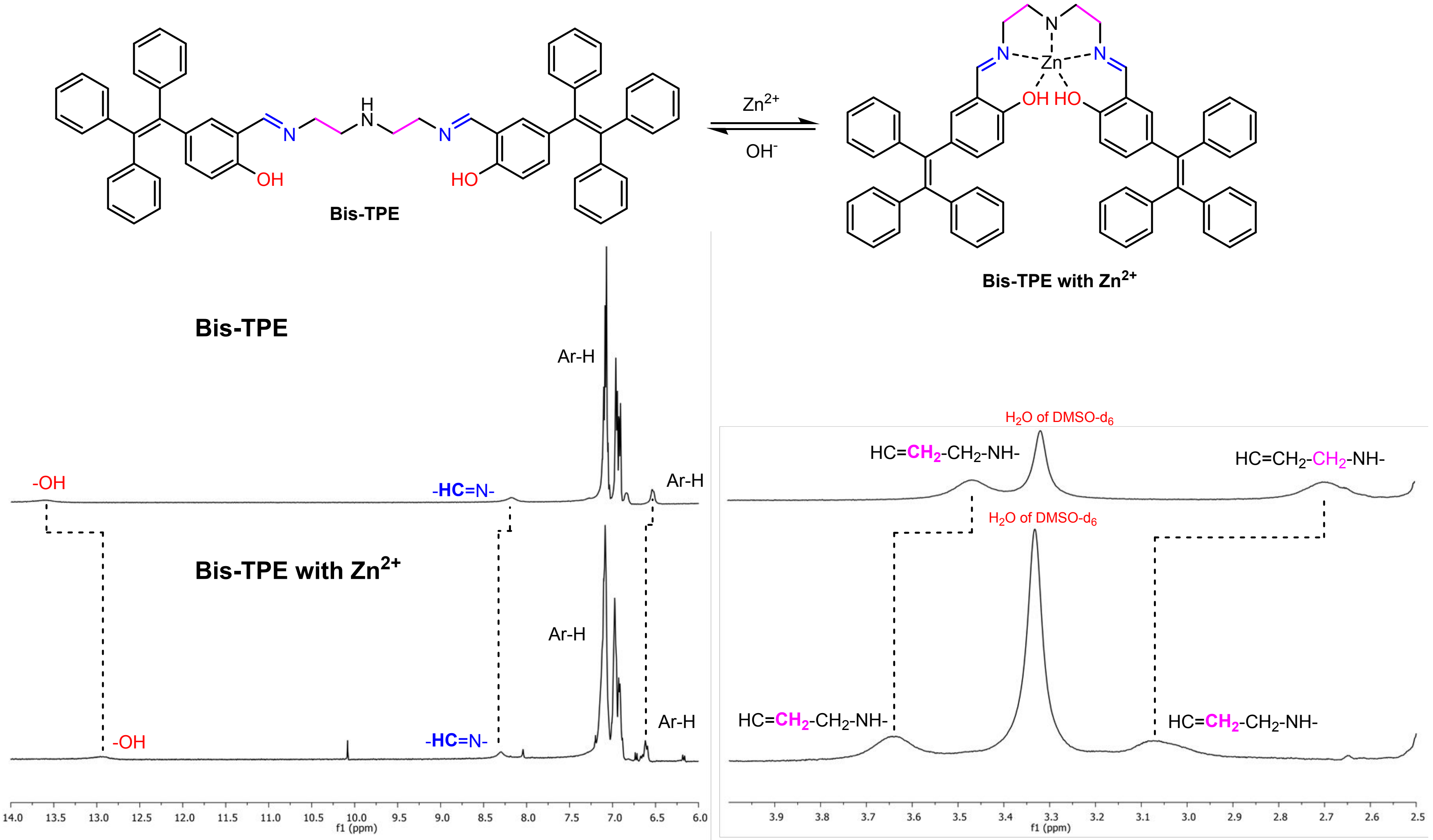

Figure S14A. The comparison of ^{1}H-NMR of Bis-TPE and Bis-TPE-Zn^{2+} in DMSO-d6.

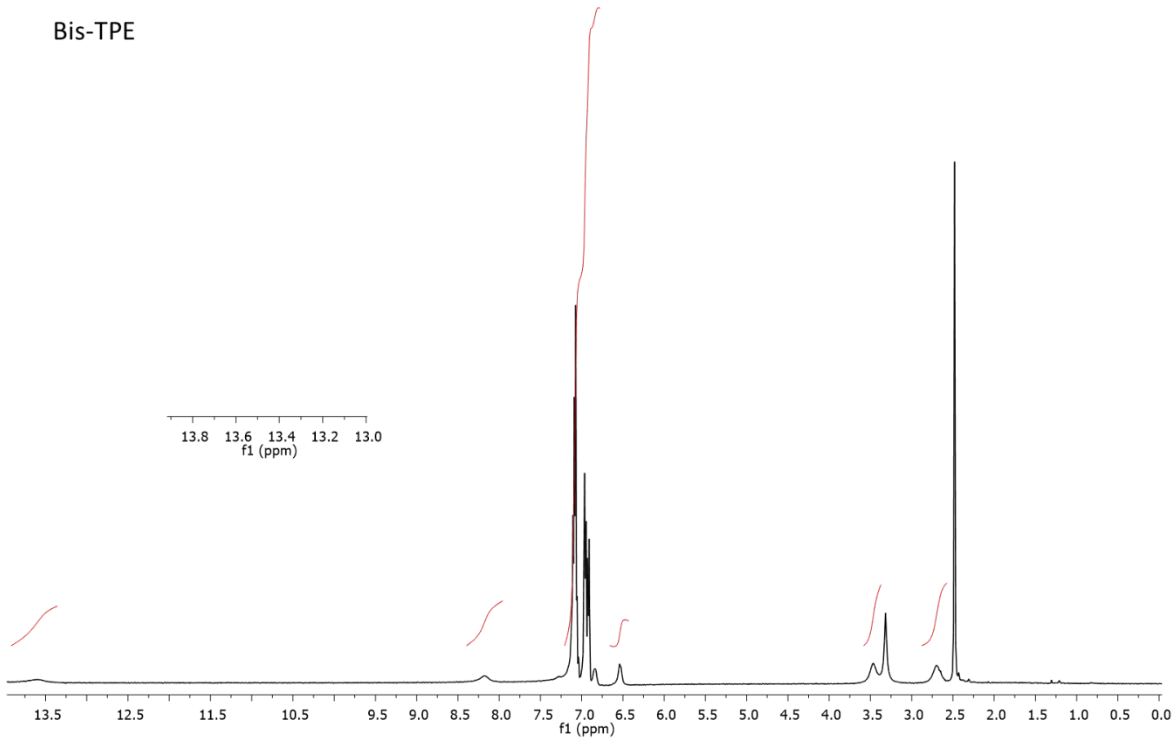

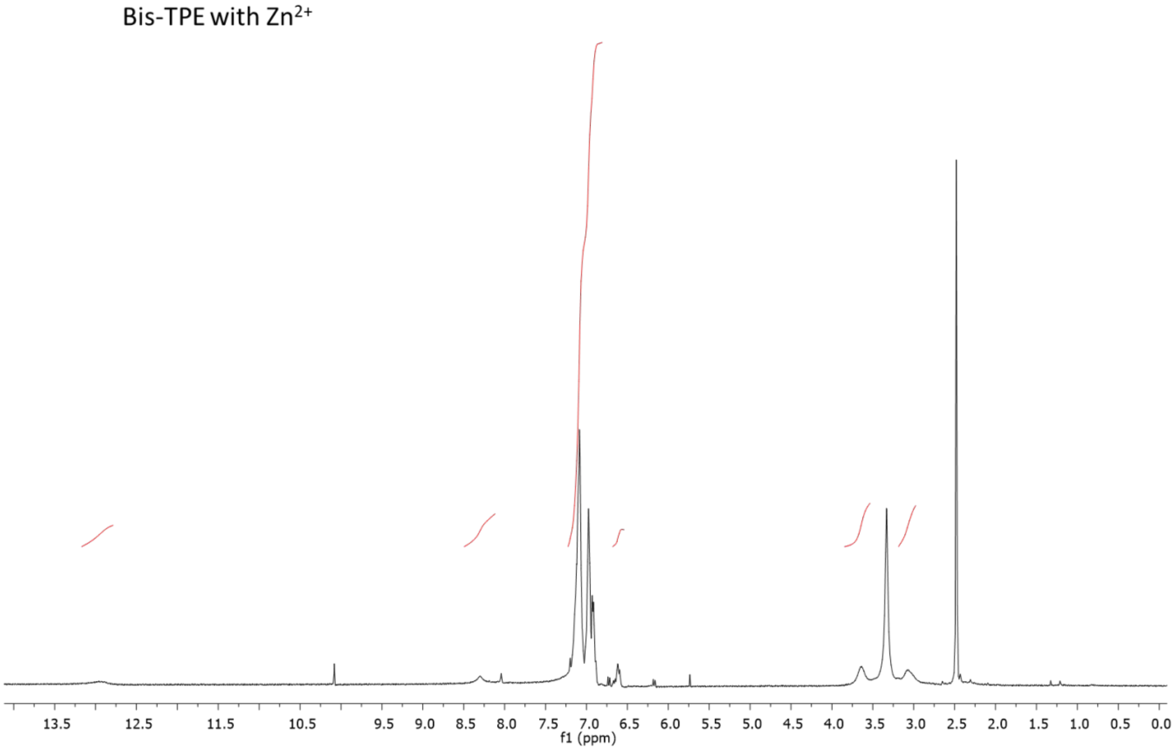

Figure S14B. The ^{1}H-NMR (400 MHz) spectrums of Bis-TPE and Bis-TPE-Zn^{2+} in DMSO-d6.

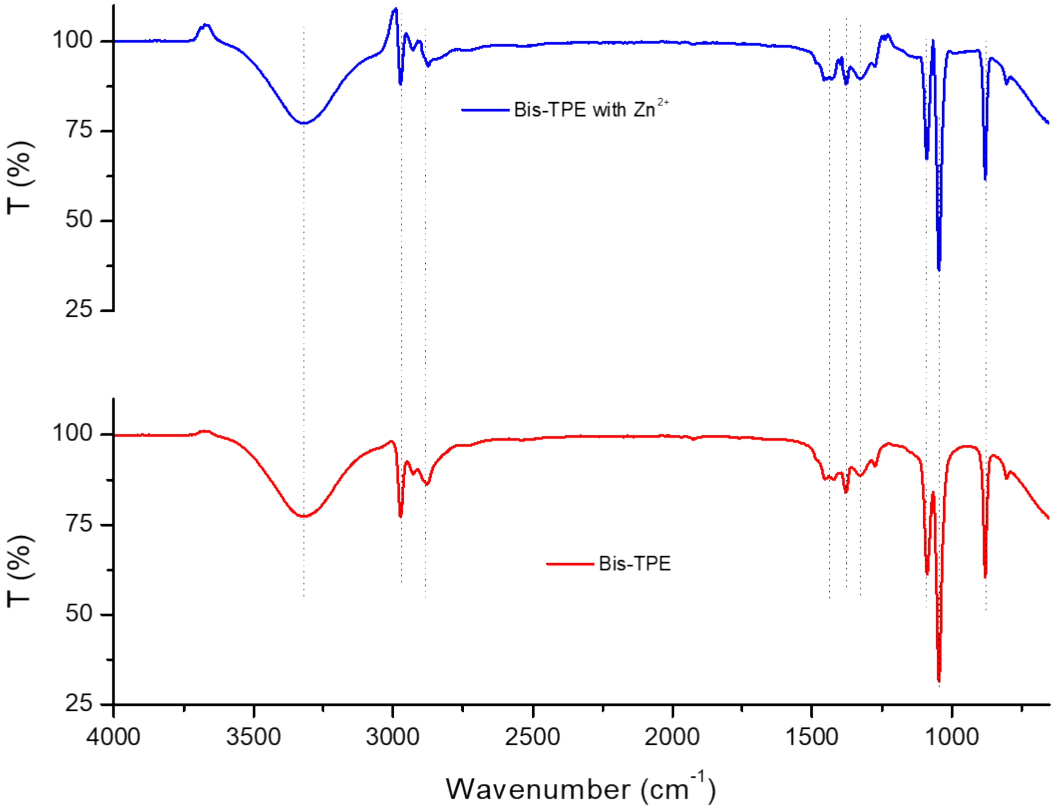

Figure S15. FTIR spectra of Bis-TPE and Bis-TPE-Zn^{2+}

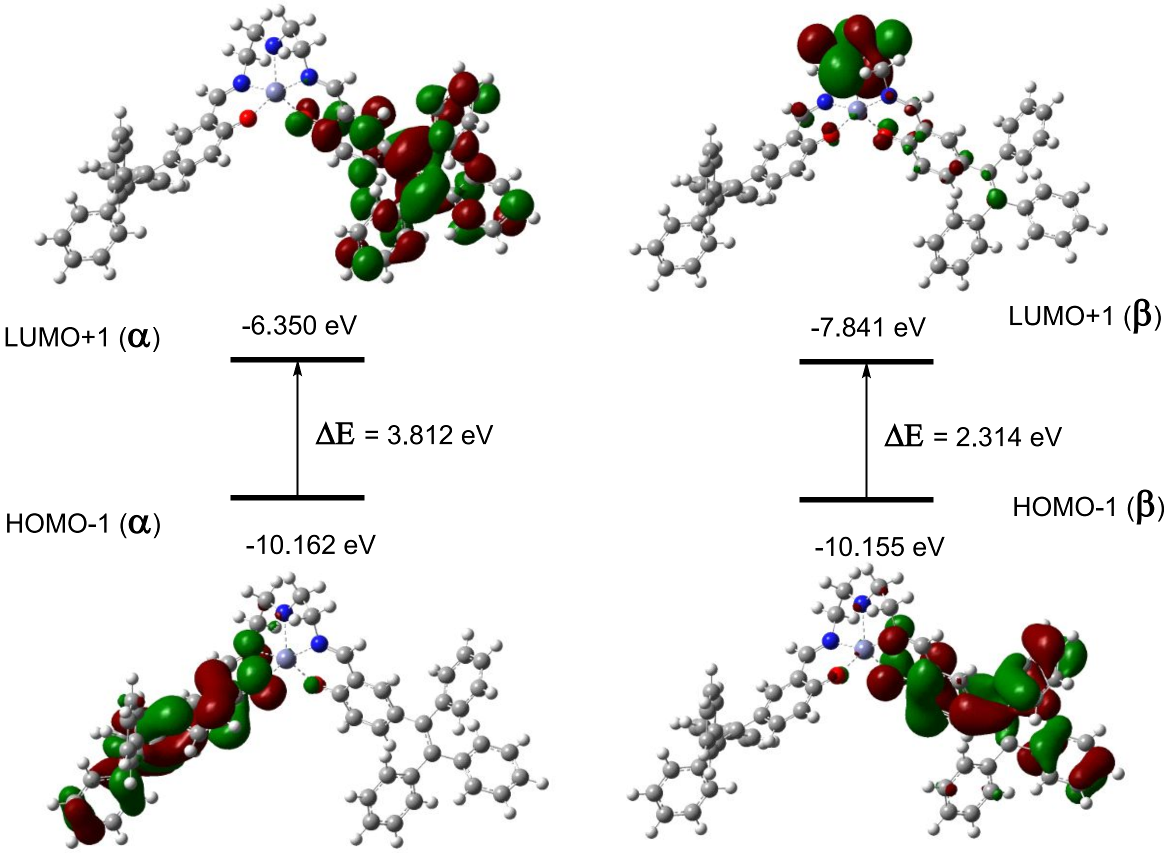

Figure S16. Diagrams of HOMO and LUMO of compound Bis-TPE–Zn^{2+}.

References

[1]. Ye, Q.; Zhu, D.; Xu, L.; Lu, X.; Lu, Q. The fabrication of helical fibers with circularly polarized luminescence via ionic linkage of binaphthol and tetraphenylethylene derivatives. J. Mater. Chem. C, 2016, 4, 1497-1503.

[2]. Feng, H. T.; Zheng, Y. S. Highly sensitive and selective detection of nitrophenolic explosives by using nanospheres of a tetraphenylethylene macrocycle displaying aggregation-induced emission. Chemistry–A European Journal, 2014, 20(1), 195-201.
